# An integrated analysis of prognostic mRNA signature in early- and progressive-stage gastric adenocarcinoma

**DOI:** 10.3389/fmolb.2022.1022056

**Published:** 2023-01-04

**Authors:** Xiaoling Hong, Kai Zhuang, Na Xu, Jiang Wang, Yong Liu, Siqi Tang, Junzhang Zhao, Zunnan Huang

**Affiliations:** ^1^ The First Dongguan Affiliated Hospital, Guangdong Medical University, Dongguan, China; ^2^ Key Laboratory of Big Data Mining and Precision Drug Design, Guangdong Medical University, Dongguan, China; ^3^ Key Laboratory of Computer-Aided Drug Design of Dongguan City, Guangdong Medical University, Dongguan, China; ^4^ Key Laboratory for Research and Development of Natural Drugs of Guangdong Province, Guangdong Medical University, Dongguan, China; ^5^ The Second School of Clinical Medicine, Guangdong Medical University, Zhanjiang, China; ^6^ School of Public Health, Guangdong Medical University, Dongguan, China; ^7^ School of Biomedical Engineering, Guangdong Medical University, Zhanjiang, China; ^8^ Department of Gastroenterology, The Sixth Affiliated Hospital of Sun Yat-sen University, Guangdong, Guangdong Institute of Gastroenterology, Guangdong Provincial Key Laboratory of Colorectal and Pelvic Floor Diseases, National Key Clinical Discipline, Guangzhou, China; ^9^ Guangdong Institute of Gastroenterology, Guangdong Provincial Key Laboratory of Colorectal and Pelvic Floor Diseases, Guangzhou, China; ^10^ Marine Medical Research Institute of Guangdong Zhanjiang, Zhanjiang, China

**Keywords:** STAD, prognostic model, mRNA, diagnosis, novel biomarkers

## Abstract

The pathogenesis and vital factors of early and progressive stages of stomach adenocarcinoma (STAD) have not been fully elucidated. In order to discover novel and potential targets to guide effective treatment strategies, a comprehensive bioinformatics study was performed, and the representative results were then validated by quantitative polymerase chain reaction (qPCR) and immunohistochemical (IMC) staining in clinical samples. A total of 4,627, 4,715, and 3,465 differentially expressed genes (DEGs) from overall-, early-, and progressive-stage STAD were identified, respectively. Prognostic models of 5-year OS were established for overall-, early-, and progressive-stage STAD, and ROC curves demonstrated AUC values for each model were 0.73, 0.87, and 0.92, respectively. Function analysis revealed that mRNAs of early-stage STAD were enriched in chemical stimulus-related pathways, whereas remarkable enrichment of mRNAs in progressive-stage STAD mainly lay in immune-related pathways. Both qPCR and IHC data confirmed the up-regulation of IGFBP1 in the early-stage and CHAF1A in progressive-stage STAD compared with their matched normal tissues, indicating that these two representative targets could be used to predict the prognostic status of the patients in these two distinct STAD stages, respectively. In addition, seven mRNAs (F2, GRID2, TF, APOB, KIF18B, INCENP, and GCG) could be potential novel biomarkers for STAD at different stages from this study. These results contributed to identifying STAD patients at high-risk, thus guiding targeted treatment with efficacy in these patients.

## 1 Introduction

Gastric cancer (GC) is one of the most common malignancies worldwide. Although many advances of systemic treatment in GC had been made over the past decades, it remained a concern that the majority of patients showed strong resistance to many treatment strategies (Rihawi et al., 2021), which resulted in a great health burden.

In GC, stomach adenocarcinoma (STAD) was the most common histological type (about 95%), and clinical guidelines had addressed differences in STAD therapeutic strategies and outcomes within different clinicopathological stages (Ajani et al., 2017). Optimally, patients with early-stage STAD undergo limited resection through endoscopies, while patients with advanced STAD require surgeries and multidisciplinary adjuvant treatments (Ajani et al., 2017). The 5-year survival rate for early-stage STAD (according to TNM malignancy classification) is 95%; however, the median survival time for patients with advanced-stage STAD was only 9–10 months (Ajani et al., 2016). Therefore, spotting high-risk STAD patients and choosing the appropriate treatment at the early time was crucial for prolonging survival time in these patients. Emerging evidence had revealed that biomarkers contributed to molecular classification, predicting prognosis, and driving precision therapy approaches in STAD population (Joshi and Badgwell, 2021). For example, Jiang et al. found that ITGB1-DT was apparently up-regulated in STAD tissues and was connected with the T stage, therapeutic effect, and poor prognosis of STAD patients, while suppression of ITGB1-DT could inhibit cell proliferation, invasion, and migration of STAD cells (Jiang et al., 2022). Furthermore, bioinformatics analysis can be used to screen key immune-related genes (IRGs) and pathways significantly linked to STAD therapy. For example, Xia et al. constructed an immune-related risk signature model consisting of BMP8A, MMP12, NRG4, S100A9, and TUBB3, which were associated with prognosis in patients and could be the potential biomarkers for immunotherapy in STAD (Xia et al., 2022). Nevertheless, the pathogenesis and vital factors of early- and progressive-stage STAD had not been fully highlighted; it is of importance to identify novel and promising targets and Cox model, elucidate the mechanism of STAD, and provide a candidate diagnosis option in patients with distinct STAD stages.

mRNA is single-stranded ribonucleic acid that carries genetic information to guide protein synthesis, and it has a central role in the pathogenesis of various cancers, including STAD. Numerous researchers underlined that mRNA had diagnostic and prognostic values in clinical practice. For example, Huang et al. demonstrated that the expression level of sirtuin-4 (SIRT4) was reduced in STAD tissues compared with normal gastric tissues and was also correlated with pathological differentiation and tumor-infiltrating depth of STAD (Huang et al., 2015). Lu et al. demonstrated that metastasis-associated lung adenocarcinoma transcript 1 (MALAT1) was up-regulated in MGC-803 cells, and the increased MALAT1 could promote the metastasis of cancer cells, while the decreased MALAT1 could suppress the progression and proliferation of STAD (Lu et al., 2019). BicC family RNA-binding protein 1 (BICC1), which codes an RNA-binding protein, proved to be significantly correlated with grade, TNM stage, invasion depth, and even immune infiltrates in STAD (Zhao et al., 2020). Taken together, these results indicated the key roles of mRNAs as the targets helping tumor diagnosis and targeted treatment.

**FIGURE 1:**
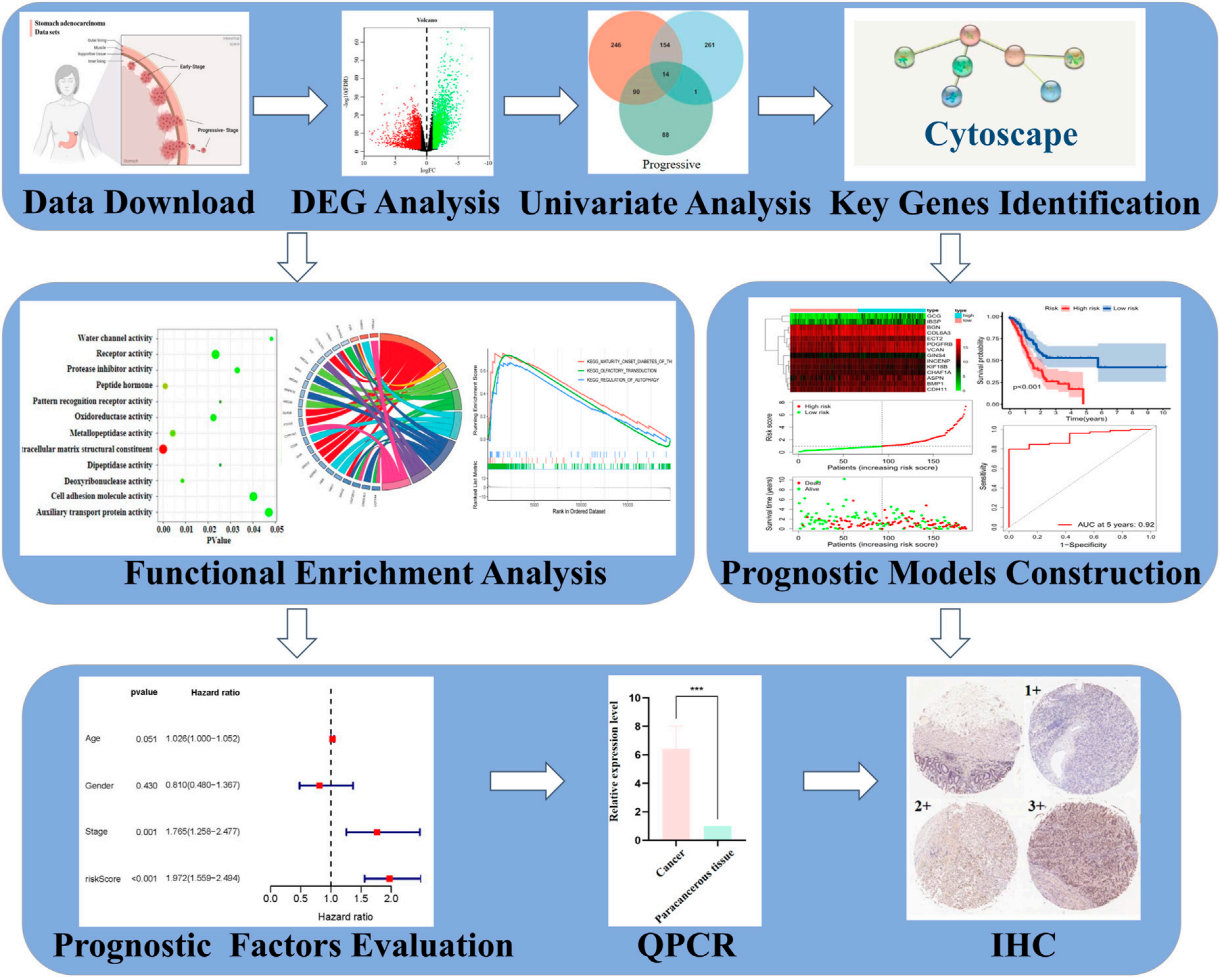
Analytic procedures of the study.

The extensive applications of gene chips and high-throughput sequencing technologies in cancer research had brought omics data explosion, and STAD is no exception. Integrated bioinformatics analysis of publicly available data of STAD improves the insight into the underlying molecular mechanism of tumorigenesis, and it also contributes to identifying potential tumor biomarkers and drug targets for STAD. Depending on these analyses in mRNAs, we distinguished STAD patients from distinct stages and accurately predicted their clinical outcomes by constructing prognostic models. Function annotations and pathway enrichment of mRNA signatures revealed that different STAD stages were dominated by distinct key mechanisms. Representative biomarkers in early and progressive stages were measured in clinical samples by qPCR and IHC detections. The overall bioinformatics analysis procedure is summarized in Figure 1. This study highlighted burgeoning evidence supporting some mRNAs as biomarkers for the diagnosis of patients in different STAD stages, which could form the basis of precision medicine strategies in the future.

## 2 Materials and methods

### 2.1 Data preparation and DEGs identification

mRNAs expression and clinical data of 407 STAD total samples were downloaded from The Cancer Genome Atlas (TCGA) database (http://portal.gdc.cancer.gov/), including 32 paracancer and 375 tumor samples. mRNAs expression and clinical data of early-stage (stages I and II) and progressive-stage (stages III and IV) STAD were classified as two independent datasets. The early-stage STAD samples included 21 paracancers and 164 tumor samples, while the progressive-stage STAD samples included 10 paracancers and 188 tumor samples. These two datasets were obtained from the 407 STAD total samples after the deletion of 24 samples without clinical staging information. All these data of STAD samples were downloaded in March 2021. Based on the three sets of STAD sample groups: overall-, early-, and progressive-stage STAD samples; differentially expressed genes (DEGs) were identified by analyzing the expression profile with edgeR R language package (Version 4.0.2) according to the cutoff criteria of *P*_*adj*_ < 0.05, |log2FC| > 1. Adjusted *p-*value took into account the false discovery rate (FDR), and the volcano maps of DEGs in these three datasets were generated.

### 2.2 Univariate Cox regression analysis

The survival package of R software was adopted to conduct the univariate Cox proportional hazard regression assessment of DEGs in the overall-, early-, progressive-stage STAD groups. With the criteria *p* < 0.05, overall survival-related mRNAs (mRNAs-OS) in each stage were obtained, which was related to the survival and prognosis of STAD patients.

### 2.3 PPI network construction and key mRNAs identification

To further identify the key mRNAs-OS in all three stages of STAD, the Search Tool for the Retrieval of Interacting Genes (STRING, http://string-db.org) was applied to analyze the mRNAs-OS and obtain protein interaction data. Proteins with a minimum required interaction score of 0.400 or above were selected to create a protein–protein interaction (PPI) network, in which the nodes with network interruption were hidden. The PPI network and its combined scores were then imported into Cytoscape software (Version 3.6.1, https://cytoscape.org/), and potential key mRNAs were identified using CytoHubba, a plug-in in Cytoscape software. According to the node degree, the top 40 candidate mRNAs selected in each stage were displayed for further analysis.

### 2.4 Establishment of three Cox proportional hazards regression models

The package “surminer” of R was applied to perform multivariate Cox proportional hazard regression analysis on those identified top mRNAs-OS, and subsequently, a prognostic model consisting of mRNAs-OS related to the patients’ prognosis (mRNAs-PRO) was constructed for the overall-, early-, progressive-stage STAD. Among them, mRNA-PRO with *p* < 0.05 was regarded as an independent prognostic factor of STAD. Based on the expression of mRNAs-PRO, the risk score of individual patients was computed as follows: risk score = Exp (mRNA_1_) × β_1_ + Exp (mRNA_2_) × β_2_ + Exp (mRNA_3_) × β_3_ +... + Exp (mRNA_n_) × β_n_. According to the median risk score, the STAD patients were clustered into high-risk and low-risk groups, and 5-year survival rates of the high- and low-risk patients were calculated for each prognostic model. Then, a risk score curve was drawn to distinguish the risk score differences between two groups of patients. A survival status map was drawn to reflect the survival status of each patient. A heatmap was drawn to exhibit the differences of the expression levels of the mRNAs-PRO in the high- and low-risk groups. A survival curve was drawn to display the 5-year survival rate in the high- and low-risk groups. An ROC curve showed by the area under the curve (AUC) of the model was drawn to evaluate its accuracy and reliability of predicting prognosis in each stage.

### 2.5 Functional enrichment and pathway analysis

To understand the underlying biological significance of mRNAs-OS in overall-stage STAD, GO function annotations including cellular component (CC), biological process (BP), and molecular function (MF) based on the FunRich (http://www.funrich.org/) database, and KEGG pathway enrichment based on KOBAS (http://kobas.cbi.pku.edu.cn/kobas3/) database were then analyzed. A *p*-value < 0.05 was set as the threshold to determine the crucial functions or pathways closely related to STAD in the overall stage. Furthermore, gene set enrichment analysis (GSEA) was carried out on the mRNAs of early- and progressive-stages STAD in order to explore the critical functions and pathways of the mRNA signatures in these two stages. The top three terms of BP, CC, MF, and KEGG pathways were presented. The threshold in this step was set based on net enrichment score (NES) and *p*-value. Gene sets with |NES| > 1 and *p* < 0.05 were considered to be statistically significantly enriched.

### 2.6 Diagnostic capability evaluation of prognostic models

In order to compare predictive accuracy of the age, gender, stage, and risk score for individual prognosis in overall-, early-, and progressive-stage STAD, the univariate Cox proportional hazards regression analysis was performed with the criterion of *Ps* < 0.05. Moreover, multivariate Cox proportional hazards regression analysis was applied to identify whether the age, gender, stage, and risk score could be independent prognostic factors in STAD patients. In addition, in order to investigate the prognostic values of mRNAs-PRO in different stratifications of other clinical prognostic variables, the overall-, early-, and progressive-stage STAD patients were clustered into different subgroups of age (≥65 or <65), gender, T stage, M stage, and N stage. The Kaplan–Meier survival curves were used to evaluate the prognostic capacity difference of three prognostic Cox models in STAD patients under different clinical variables.

### 2.7 Quantitative real-time polymerase chain reaction and immunohistochemical staining

A total of 30 pairs of cDNA tissue chips (including 13 early-stage pairs and 17 progressive-stage pairs), 84 pairs of tissue microarrays (including 32 early-stage pairs and 52 progressive-stage pairs), and the related clinicopathological information of these matched STAD and normal samples were obtained from Shanghai OUTDO Biotech Co., Ltd. (Shanghai). All patients were pathologically diagnosed as STAD according to American Joint Committee on Cancer (AJCC) criteria. The samples were obtained following written consent in accordance with an established protocol approved by Institutional Review Board of Biobank in Shanghai Outdo Biotech Co., Ltd.

QPCR was used to detect the expression levels of insulin-like growth factor binding protein-1 (IGFBP1) and chromatin assembly factor 1 subunit A (CHAF1A) in 30 pairs of cancer and normal samples (detailed clinical data are seen in Supplementary Table S1), and GAPDH was used as a reference gene. Total RNA was extracted from tissues and cells by the TRIzol reagent (Sigma, America). The cDNA was obtained by reverse transcription of RNA using PrimeScript^TM^ RT Master Mix (perfect real time) (Takara, Japan) according to the protocol of manufacturer. The expressions of IGFBP1 and CHAF1A were determined by the protocol of the ChamQ Universal SYBR qPCR Master Mix (Vazyme, Nanjing, China), according to the manufacturer’s protocol. The primers used in this study were as follows: IGFBP1, forward: 5′-GCA​TTT​CTG​CTC​TTC​CAA​AG-3′, reverse: 5′-GCA​ACA​TCA​CCA​CAG​GTA​G-3’; and CHAF1A, forward: 5′-AAA​GGA​GCA​GGA​CAG​TTG​GA-3′, reverse: 5′-CTG​GAA​GGG​ACT​TGA​TTT​GC-3’.

IHC was conducted to detect the protein levels of IGFBP1 and CHAF1A in 84 pairs of cancer and normal samples (detailed clinical data are seen in Supplementary Table S2), according to standard procedures by two independent pathologists blinded to the study. Based on proportion and staining intensity of positive stained cells, expression levels of IGFBP1 and CHAF1A were accessed semi-quantitatively (Proteintech, China). Proportion was evaluated using semi-quantitative criterion: 0, (no staining); 1, minimal (< 10%); 2, moderate (10–50%); and 3, diffuse (> 50%) staining cells. Staining intensity was also scored as 0 (negative); +1 (weak); +2 (moderate); and +3 (strong). Taken together, the final expression score of each case, considering both proportion and staining intensity, was given as 0+ (0), negative; 1+ (1 or 2), weakly positive; 2+ (3 or 4), moderately positive; and 3+ (5 or 6), strongly positive. The statistical analysis was performed using a software package (SPSS, version 19.0, Chicago, IL, United States). Clinical pathological features and expression data of representative targets were analyzed using Pearson’s chi-square and likelihood ratio tests. A level of *p* < 0.05 was considered statistically significant.

## 3 Results

### 3.1 Differential expression analysis

**FIGURE 2:**
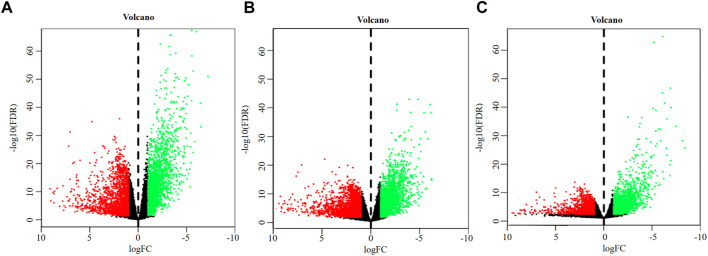
Volcano plots of DEGs. **(A)** DEGs of overall-stage STAD group. **(B)** DEGs of the early-stage STAD group. **(C)** DEGs of the progressive-stage STAD group. The abscissa denotes the log_2_ transformation value of the differential expression fold change between the STAD samples and the matched paracancerous samples. The ordinate denotes the -log_10_ transformation value of the *P*_*adj*_ (FDR) value. Green dots symbolize significantly downregulated mRNAs. Red dots symbolize significantly upregulated mRNAs.

A total of 4,627 DEGs were identified from the overall stage of STAD, composed of 2,445 up-regulated mRNAs and 2,182 down-regulated mRNAs (Figure 2A; Supplementary Table S3). In addition, 4,715 DEGs were distinguished from early-stage STAD, in which 2,542 DEGs were remarkably up-regulated and 2,173 DEGs were notably down-regulated (Figure 2B, Supplementary Table S3). A total of 3,465 DEGs were detected from progressive-stage STAD, composed of 1,493 DEGs up-regulated mRNAs and 1972 down-regulated mRNAs (Figure 2C, Supplementary Table S3).

**FIGURE 3:**
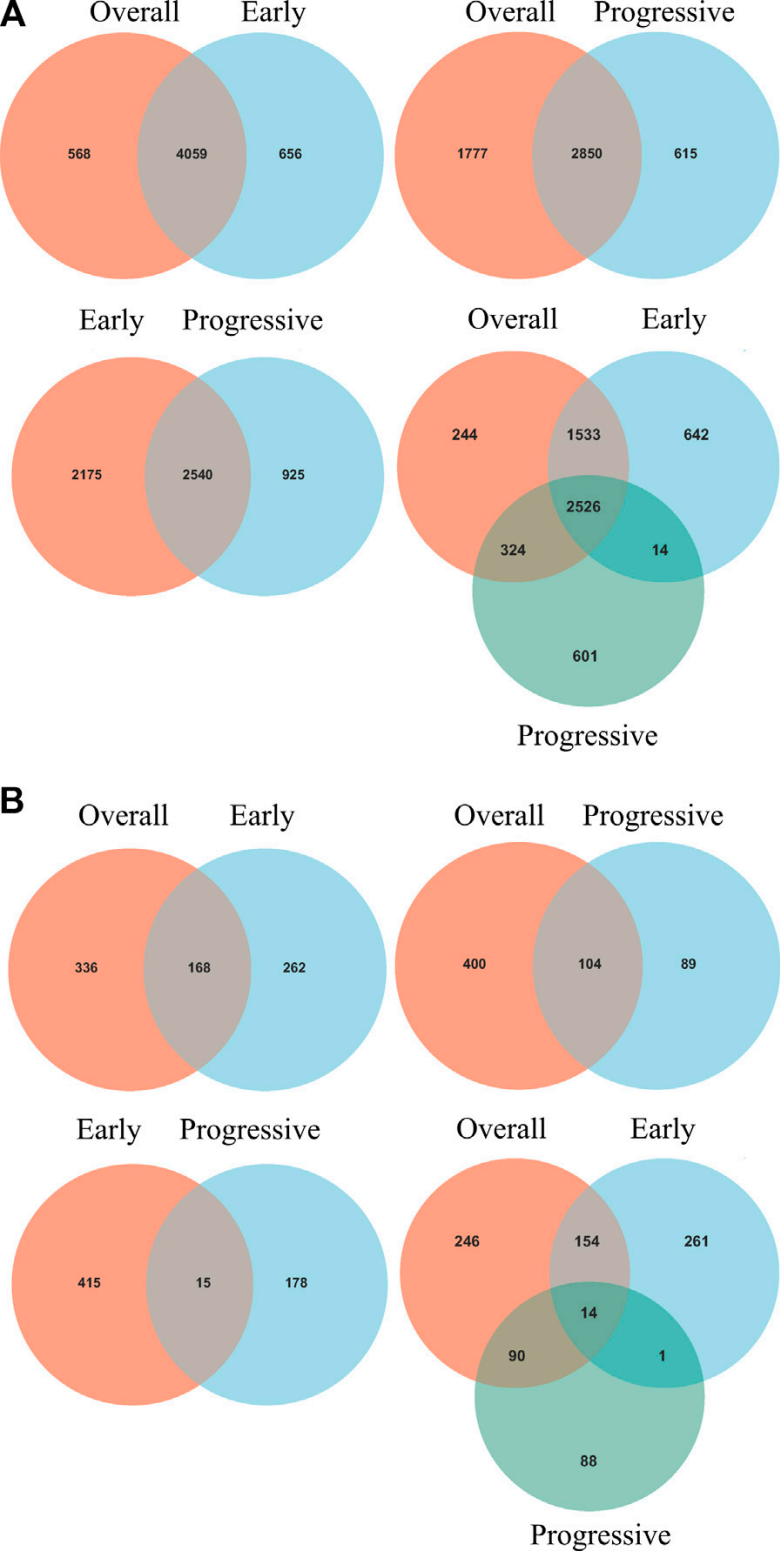
Venn diagrams of overlapping DEGs and mRNAs-OS from overall-, early-, and progressive-stage STAD. Venn diagrams are shown for overlapping DEGs **(A)** or mRNAs-OS **(B)**. Terms over the circles represent the groups of DEGs or mRNAs-OS at different stages of STAD.

Intersections between overall- and early-stage, overall- and progressive-stage, and early- and progressive-stage STAD provided 4,059, 2,850, and 2,540 overlapping signatures, respectively. Overlapping of target DEGs of all three stages in STAD obtained 2,526 consensus mRNAs (Figure 3A, Supplementary Table S4).

Interestingly, six mRNAs, including SCGB3A1, SRARP, MUC5B, GABRB1, CNMD, and KRT27, were all up-regulated in early-stage STAD but down-regulated in progressive-stage STAD, while these targets were not significantly different when brought into overall STAD samples (Supplementary Table S4).

### 3.2 Cox proportional hazards regression model of DEGs

Univariate Cox regression analysis identified 504, 430, and 193 mRNAs-OS for the overall-, early-, and progressive-stage STADs (Supplementary Table S5). Intersections analyzes revealed 168, 104, and 15 overlapping mRNAs-OS between overall- and early-stage STAD, overall- and progressive-stage STAD, and early- and progressive-stage STAD, respectively (Figure 3B, Supplementary Table S6). Moreover, after overlapping mRNAs-OS among all three stages of STAD, a total of 14 signature genes were obtained (Figure 3B, Supplementary Table S6). In addition, CytoHubba plug-in analysis selected 40 top mRNAs-OS from the overall-, early-, and progressive- stage STAD (Supplementary Table S7).

**FIGURE 4:**
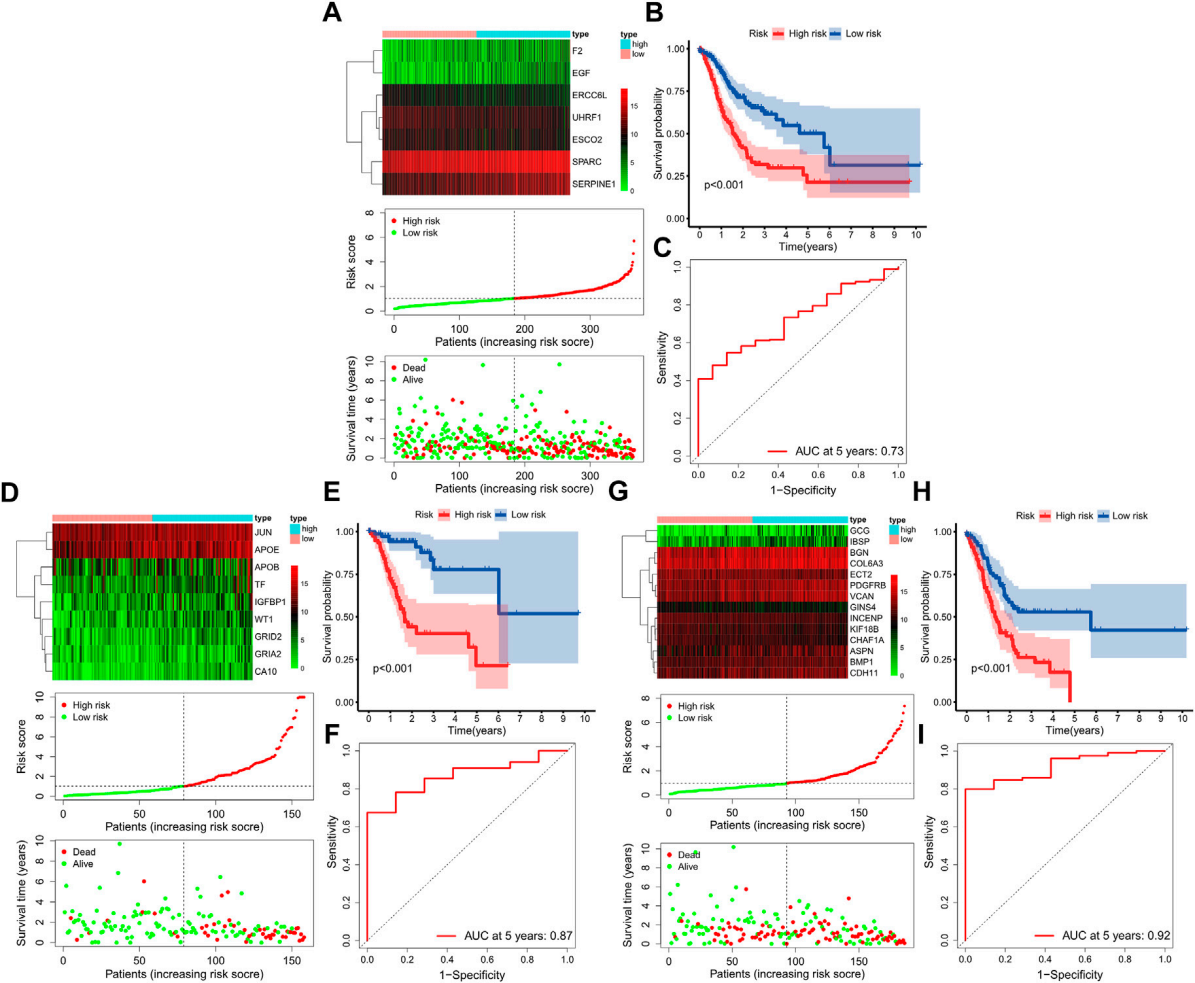
Construction of three prognostic models. The top, bottom left, and bottom right corner of this figure (three subgraphs) represent the STAD total samples group **(A–C)**, STAD samples in the early-stage group **(D–F)**, and STAD samples in the progressive-stage group **(G–I)**. **(A,D,G)** From top to bottom of every subgraph are the expression heatmap, risk score curve, and survival status map between the low-risk and high-risk groups. The color bar reveals the relative mRNAs-PRO expression level, with red denoting high expression and green denoting low expression. **(B,E,H)** Survival curve for the low-risk and high-risk groups. **(C,E,I)** ROC curve for survival predictions.

Further multivariate Cox analysis based on those top mRNAs-OS constructed three prognostic models with 7, 9, and 14 mRNAs-PRO for these three stage sets (Figure 4, Supplementary Table S8). The survival risk score based on mRNAs-PRO for each stage was calculated by the model formula and stratified patients into low- and high-risk groups based on their median risk score. As presented in Figures 4A,D,G, expression heatmaps, risk score curve, and survival status map were plotted between the low- and high-risk groups of 7-, 9-, and 14-mRNA-based prognostic models for the overall-, early-, and progressive-stage STAD, respectively. The Kaplan–Meier survival curve of the high-risk group and the low-risk group for overall-, early-, and progressive-stage STAD analysis is presented in Figures 4B,E,H. The AUC value of the ROC curve was 0.73, 0.87, and 0.92 for overall, early-, and progressive-stage STAD, respectively, indicating that these models could form reliably to accurately predict the prognosis of STAD patients (Figures 4C,F,I).

### 3.3 Functional enrichment analysis

**FIGURE 5:**
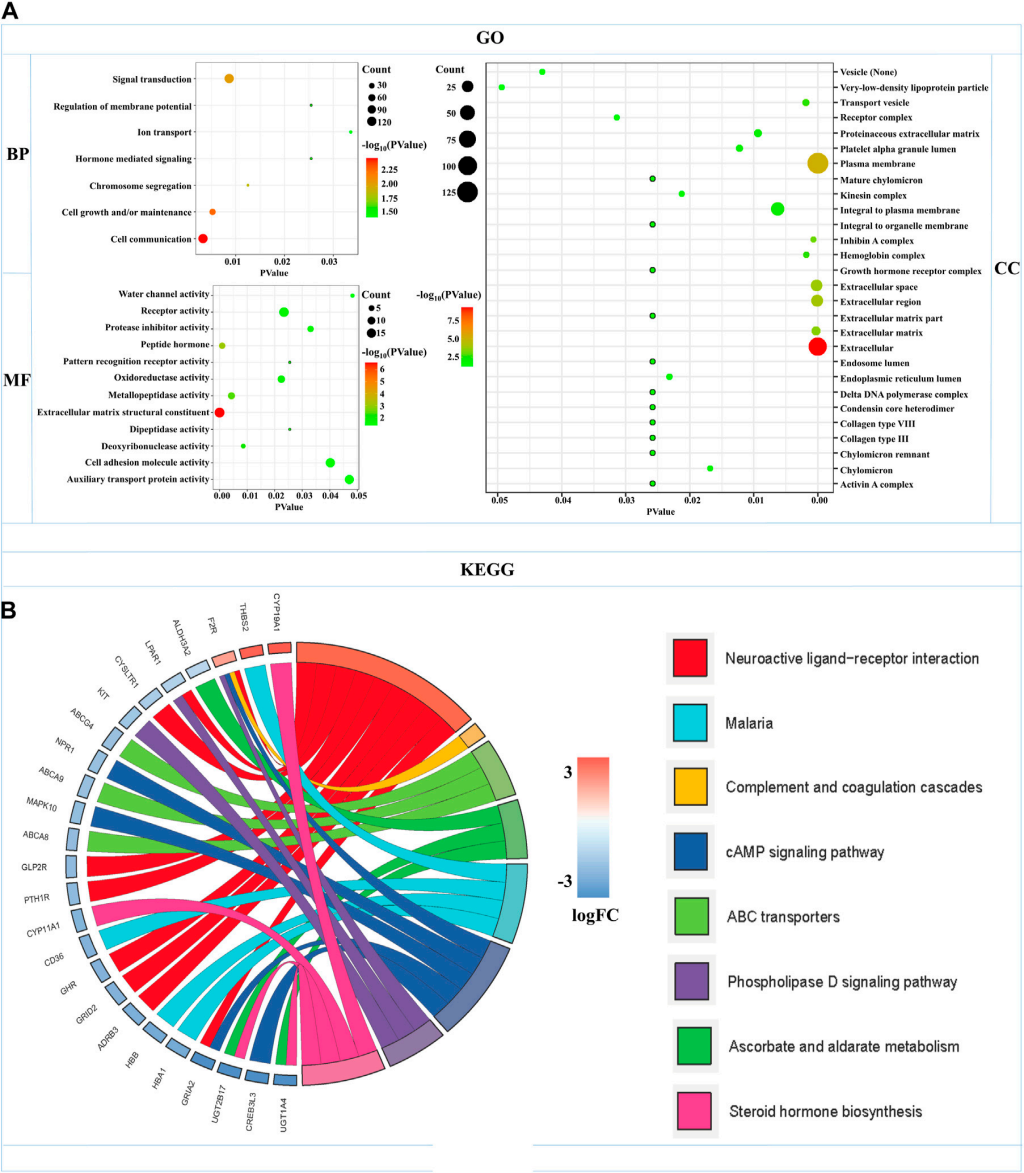
Functional enrichments of mRNAs-OS in overall-stage STAD. **(A)** GO function annotations (including BP, CC, and MF). The abscissa represents the *p*-value, and the ordinate represents the name of the functions. The bubble area increases with the number of mRNA-OS increasing. **(B)** KEGG pathways analysis. The color of the columns changes from blue to red as the degree of mRNAs enriched in the pathways increases.

GO functional annotation and KEGG pathway analyses were carried out by the FunRich and KOBAS databases in 504 mRNAs-OS of the overall-stage STAD. mRNAs-OS of overall stage were enriched in 47 GO terms (*p* < 0.05), composed of seven BP, 12 MF, and 28 CC items. As exhibited in Figure 5A, in BP, the smallest *p*-value lay in the cell communication item (*p* = 3.27E-3), while annotation with the largest count number annotation was signal transduction (count = 122). The annotation with the smallest *p*-value and largest count number in the MF category was the extracellular matrix structural constituent (*p* = 2.54E-7, count = 18). As for CC, mRNAs-OS was remarkably enriched in functions as extracellular (*p* = 5.68E-10), and the largest count number was laid in the plasma membrane (count = 127). KEGG pathway analysis demonstrated that enriched mRNAs-OS were notably involved in 68 pathways, and the top eight pathways with smallest *p*-values are shown in Figure 5B, including neuroactive ligand–receptor interaction, complement and coagulation cascades, ABC transporters, ascorbate and aldarate metabolisms, malaria, cAMP signaling pathway, phospholipase D signaling pathway, and steroid hormone biosynthesis.

**FIGURE 6:**
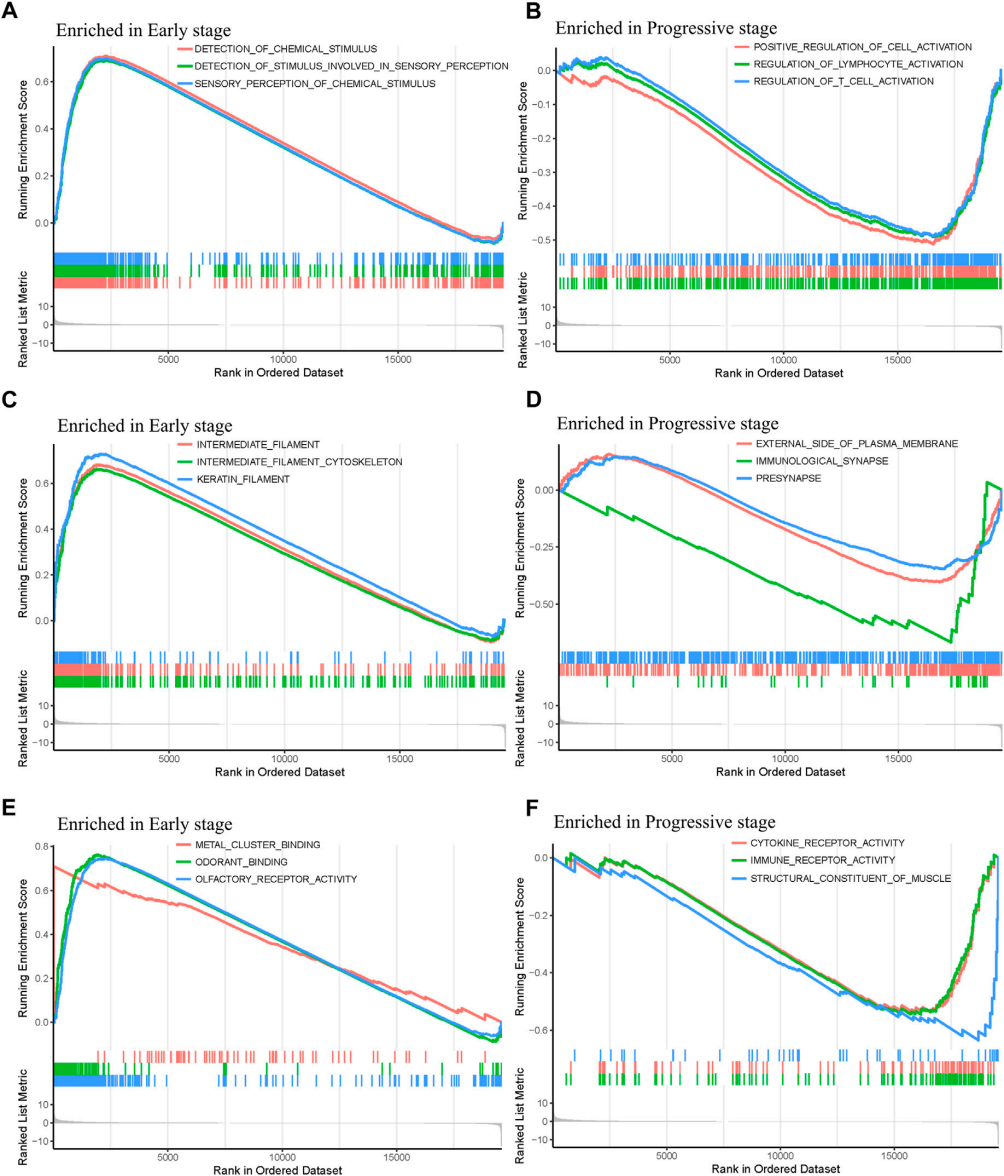
GO function annotations of mRNAs in the early- and progressive-stage STAD cohorts *via* GSEA. The left side of the figure refers to the enriched annotations in the early-stage group, while the right side refers to the enriched annotations in the progressive-stage group. From top to bottom of this figure are biological process (BP) **(A,B)**; cellular component (CC) **(C,D)**; and molecular function (MF) **(E,F)**

**FIGURE 7:**
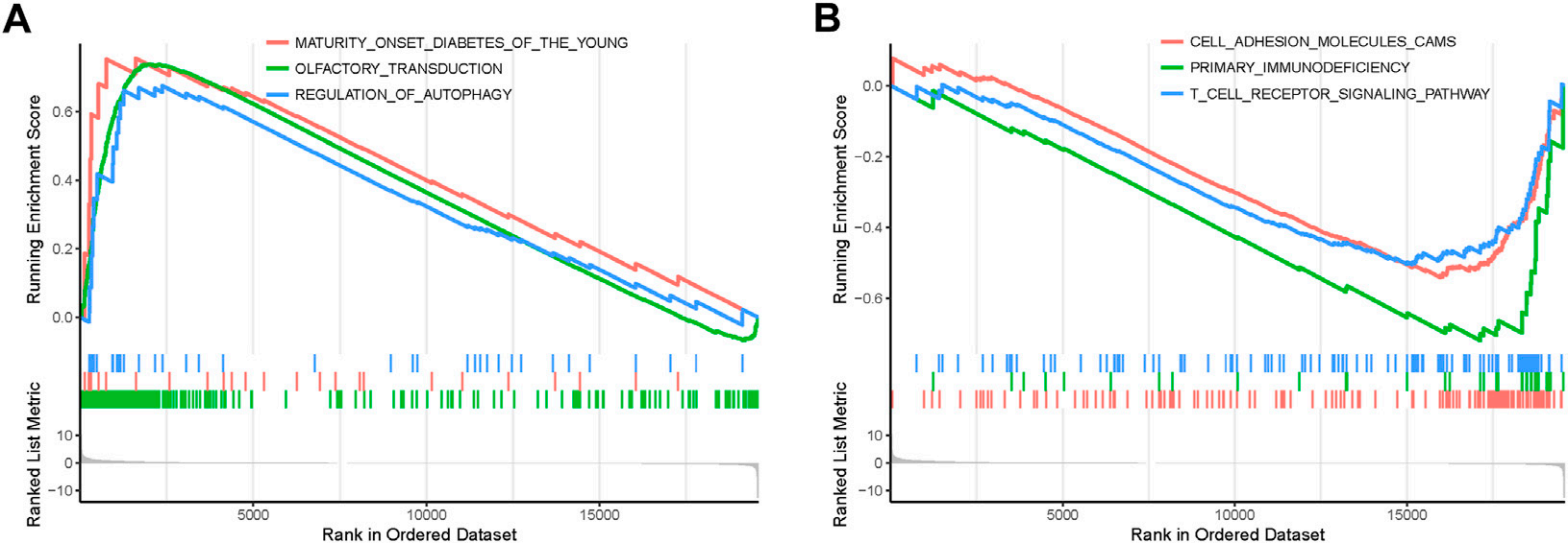
KEGG pathways of mRNAs in the early- and progressive-stage STAD cohorts *via* GSEA. **(A)** Enriched pathways in early-stage STAD. **(B)** Enriched pathways in progressive-stage STAD.

In order to explore the functions and pathways of mRNAs in different stages of STAD, gene set enrichment analysis (GSEA) was conducted in early- and progressive-stage STAD, respectively. Three most significantly enriched pathways were exhibited in Figure 6. As for BP, enrichment of mRNAs was remarkably laid in detection of chemical stimulus, detection of stimulus involved in sensory perception, and sensory perception of chemical stimulus in early-stage STAD (Figure 6A), while those mRNAs were significantly enriched in positive regulation of cell activation, regulation of lymphocyte activation, and regulation of T cell activation in progressive-stage STAD (Figure 6B). mRNAs of CC were markedly enriched in intermediate filament, intermediate filament cytoskeleton, and keratin filament in early-stage STAD (Figure 6C), while they were notably enriched pathways in progressive-stage STAD including external side of plasma membrane, immunological synapse, and presynapse (Figure 6D). The significantly enriched pathways in MF included olfactory receptor activity, odorant binding, and metal cluster binding in early-stage STAD (Figure 6E), while pathways of progressive-stage STAD enriched in immune receptor activity, cytokine receptor activity, and structural constituent of muscle (Figure 6F). In addition, KEGG pathway analysis of early-stage STAD revealed that mRNAs were enriched significantly in olfactory transduction, maturity onset diabetes of the young, and regulation of autophagy (Figure 7A), while mRNAs were enriched in pathways including cell adhesion molecules cams, primary immunodeficiency, and T-cell receptor signaling pathway in progressive-stage STAD (Figure 7B).

### 3.4 Prognostic model validation

**FIGURE 8:**
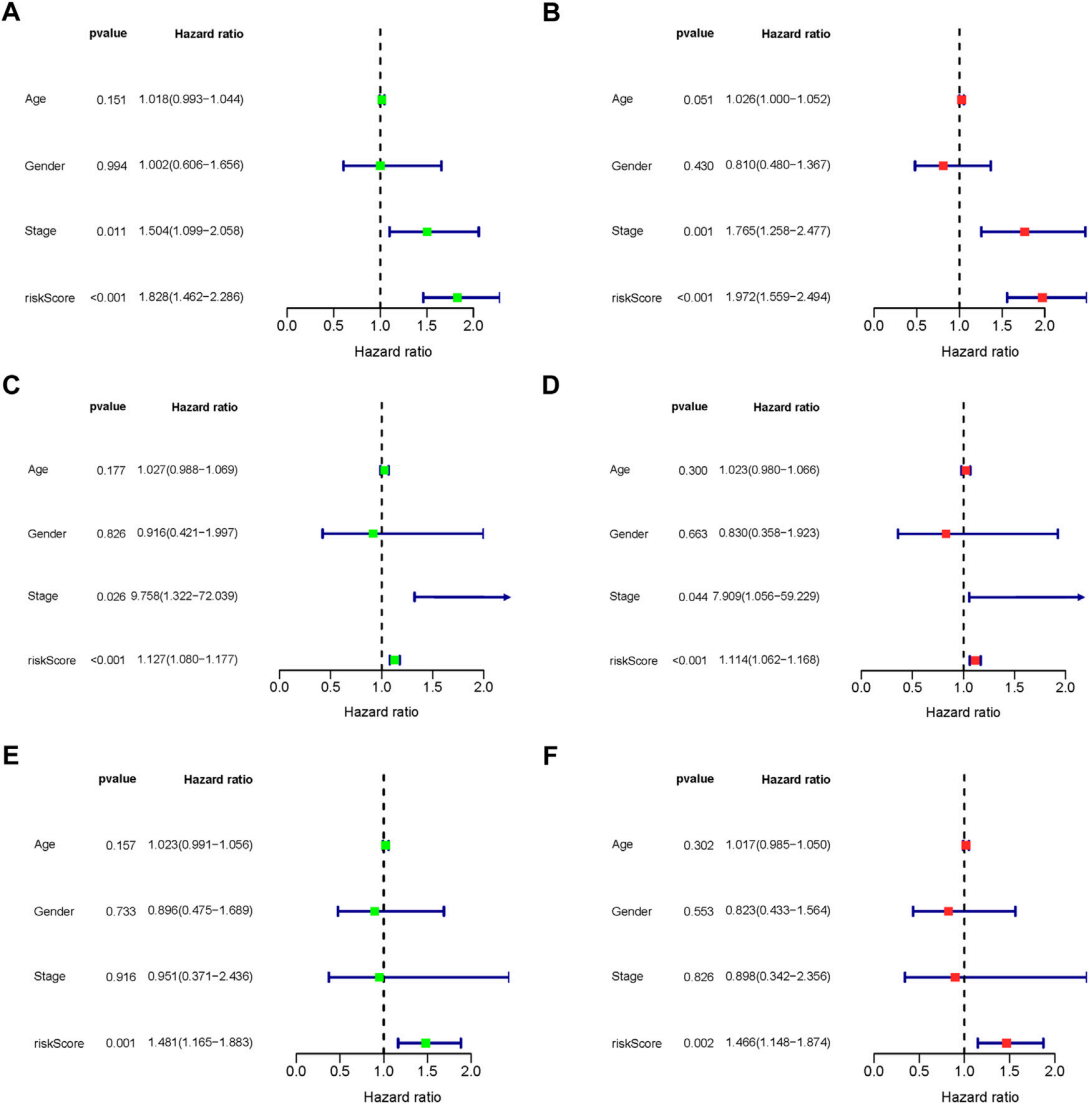
Forest plot of clinical factors prognostic analysis. **(A–B)** Overall-stage STAD; **(C–D)** early-stage STAD; and **(E–F)** progressive-stage STAD. **(A,C,E)** Univariate analysis of clinical factors. **(B,D,F)** Multivariate analysis of clinical factors. The dotted line indicates risk ratio (HR) = 1, the green or red rectangle to the left of the dotted line indicates that the clinical factor is a protective factor for STAD (HR < 1), and the green or red rectangle to the right of the dotted line indicates that the clinical factor is a risk factor for STAD (HR > 1).

The predictive association between the age, gender, stage and risk score, and individual prognosis was evaluated by univariate and multivariate Cox regression analyses. Figure 8 shows both univariate and multivariate Cox regression analyses of overall survival time in overall-, early-, and progressive-stage STAD patients. As exhibited in the forest plots, while stage were significantly associated with the overall survival time in overall- and early-stage STAD patients (*Ps* ≤ 0.044) (Figures 8A–D), the risk score was the only independent factor for all the three stages STAD patients by both univariate and multivariate Cox regression models (*Ps* ≤ 0.002) (Figures 8A–F). These demonstrated the rationality of the stage stratification in the overall group and the risk-based prognostic models built for the overall-, early-, and progressive-stage STAD patients in this study. Moreover, the reliability and validity of prognostic models in classifying the high- and low-risk groups were further confirmed under various clinical circumstances by the Kaplan–Meier survival curves, and the overall survival (OS) time in the high-risk group was significantly lower than that of the low-risk group in overall- (Supplementary Figure S1), early- (Supplementary Figure S2), and progressive- (Supplementary Figure S3) stage STADs in all clinical situations, including age (≥ 65 or < 65), gender, T stage, M stage, and N stage (all *p* < 0.05). Thus, the prognostic models of overall-, early-, and progressive-stage STADs could be well used in a variety of clinical practices, indicating these three prognostic models were reliable and stable under different clinical circumstances.

### 3.5 QPCR and IHC of IGFBP1 and CHAF1A in STAD samples

**FIGURE 9:**
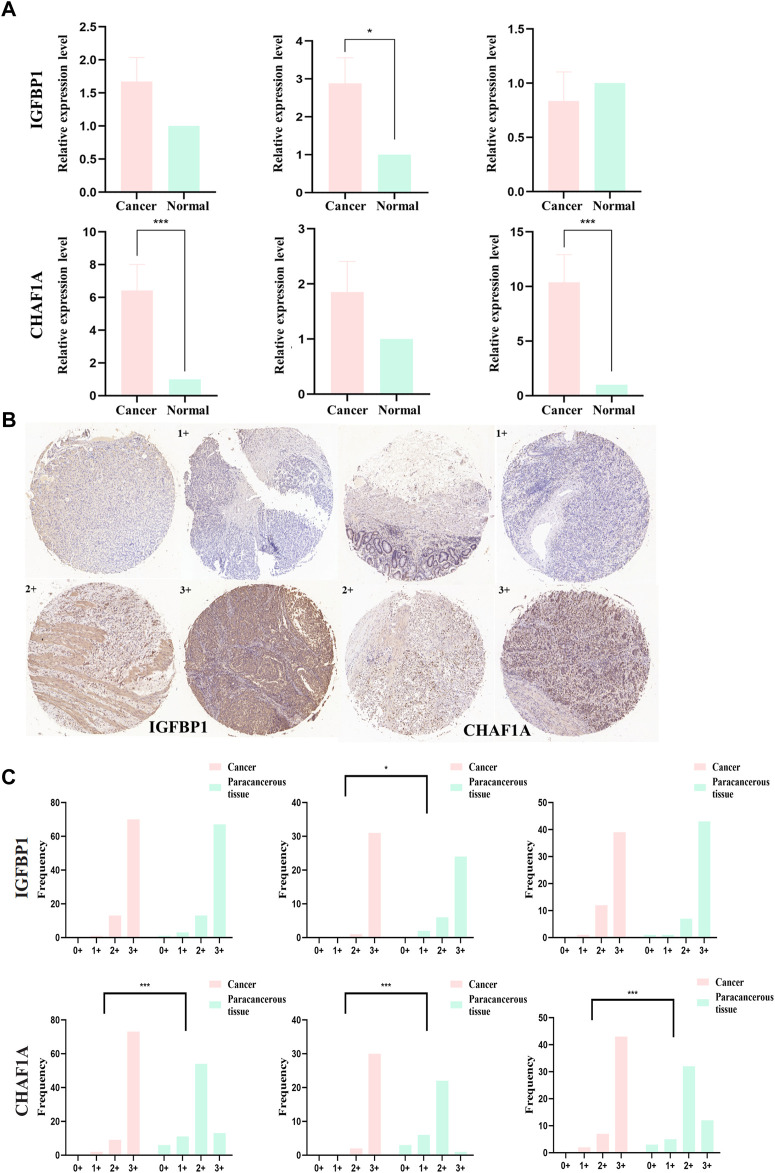
mRNA and protein expression levels of IGFBP1 and CHAF1A evaluated from STAD and normal samples by qPCR and IHC, respectively. **(A)** MRNA expression levels of IGFBP1 and CHAF1A detected by qPCR in 30 STAD and paired paracancerous normal tissues (early-stage: 13 pairs and progressive-stage: 17 pairs). **(B)** Representative images of IHC displaying negative (0), low (1+), moderate (2+), and strong (3+) staining for IGFBP1 and CHAF1A proteins from 84 STAD and paired paraneoplastic normal tissues (early-stage: 32 pairs and progressive-stage: 52 pairs). Various staining intensities of matched cancer and normal tissues were demonstrated, respectively, for IGFBP1 and CHAF1A. **(C)** IHC scores of overall, early-stage, and progressive-stage STAD, and paired normal tissues are presented in histograms with frequency. The statistical analysis was performed using Pearson’s χ2 test and likelihood ratio test. *p < 0.05 and ***p < 0.001.

Relative mRNA levels of IGFBP1 and CHAF1A in STAD and matched paracancerous tissues were evaluated *via* qPCR in overall-, early-, and progressive-stage STAD (Figure 9A), and the results showed the expression of IGFBP1 was significantly elevated in early-stage STAD in comparison with matched normal tissues (*p* = 0.018, Figure 9A-top), which was in line with bioinformatics findings. CHAF1A was markedly increased in overall- and progressive-stage STAD in comparison with matched normal tissues (both *p* < 0.001, Figure 9A-bottom), which was also consistent with expected analyses.

In addition, Figure 9B showed the representative IHC staining on the protein levels of IGFBP1 and CHAF1A, and Figure 9C demonstrated the cytoplasmic immunoreactivity of these two representative targets in the paired cancer and paraneoplastic normal tissues of STAD patients. For IGFBP1, cancer samples demonstrated stronger staining than matched normal tissues in early-stage STAD (*p* = 0.022, Figure 9C-top). For CHAF1A, cancer samples showed much stronger staining than matched normal tissues in the overall-, early-, and especially the progressive-stage STAD (all *p* < 0.001, Figure 9C-bottom). The IHC results on the protein levels of these two representative targets were basically lined well with the qPCR results on their mRNA expressions; both were in accordance with the calculation results. Thus, IGFBP1 could serve as a biomarker for early-stage STAD, while CHAF1A could act as a biomarker for progressive-stage STAD.

## 4 Discussion

According to the Global Cancer Statistics 2020, the incidence and mortality of GC ranks the fifth and fourth in all malignancies, respectively (Sung et al., 2021). It causes a considerable global health burden and requires urgent attention (Yue et al., 2021). STAD accounts for 90% of gastric cancer and is the most common histological type of this malignance (Joshi and Badgwell, 2021). As the onset of STAD is insidious, most patients had already developed an advanced stage of cancer when they were confirmed diagnosis (Joshi and Badgwell, 2021). Current interventions have highlighted advances in therapeutics of STAD, including surgery, systemic chemotherapy, radiotherapy, immunotherapy, and targeted therapy; however, the efficacy does not meet the need for advanced gastric cancer (Jim et al., 2017), and the 5-year survival rate of distant-stage gastric cancer is less than 10% (Li et al., 2022). Under the passive situations nowadays, discovery of novel diagnostic biomarkers and biomarker-driven therapy for STAD is urgently needed.

With the development of high-throughput sequencing technologies, the mRNA molecules have been emerging as a new class of cancer biomarkers. Recent studies have explored associations between mRNAs and cancer features, and moreover, various mRNA signatures have been reported in STAD for prognostic analysis (Ren et al., 2020; Wu et al., 2020; Zhou et al., 2020). However, some limitations of these studies should be noticed: most of them were reports of key biomarkers in overall STAD, while neglecting STAD stages (Li et al., 2019; Fu et al., 2020; Qiu et al., 2020). Nevertheless, STAD is a cancer characterized with high heterogeneity, and the differences of pathological features in distinct STAD stages were evident (Ren et al., 2021). Therefore, it is inappropriate to use the same mRNAs as biomarkers to classify patients with STAD at all the different stages in the clinical work.

This study identified 4,715 DEGs of early-stage STAD and 3,465 DEGs of progressive-stage STAD, and a total of 2,540 DEGs were overlapping, indicating that many remaining DEGs were evidently different in early- and progressive-stages STAD, and these DEGs might be vital to characterize the two stages. Interestingly, six mRNAs (SCGB3A1, SRARP, MUC5B, GABRB1, CNMD, and KRT27) were up-regulated in early-stage STAD, while they were down-regulated in progressive-stage STAD. This suggested these mRNAs might play a role in different stages of cancers by participating in different mechanisms; meanwhile, these signatures might act as convenient biomarkers to distinguish STAD stages.

Multivariate Cox regression analysis of the age, gender, stage, and risk score demonstrated that the tumor stage was an independent prognostic factor, and the mRNAs-PRO signatures for distinct STAD stages were potential predictors for prognosis.

The prognostic models of overall-, early-, and progressive-stage STAD were, respectively, composed of 7 mRNAs, 9 mRNAs, and 14 mRNAs. There was no consensus mRNAs-PRO in these three prognostic models. The AUC values of ROC curves of the prognostic models for overall, early, and progressive groups were 0.73, 0.87, and 0.92, respectively. The value of the overall stage was the smallest, suggesting a sorting stage might slightly improve the AUC value and the predictive reliability of the prognostic models. We also constructed a prognostic model by analyzing the data of all GC patients with the same procedure as the other three sets of data, and the value of the AUC was only 0.70, which was worst (data were not shown). This re-emphasized that the prognostic model established by stratified data according to influential clinical variables was more effective. As the amount of experimental data increased, novel molecular biomarkers could be identified to facilitate more accurate predictions after stratification. This might indicate that the prognostic model constructed for the overall-stage STAD was problematic due to the stage mixing.

Table 1 compared the abnormal expression of the prognostic mRNAs-PRO predicted in this study with those reported in previous studies. In the prognostic model of overall-stage STAD, all seven mRNAs (EGF, SERPINE1, ESCO2, ERCC6L, UHRF1, SPARC, and F2) were calculated to be up-regulated in this study. After a systematic and careful literature research, we found that the former six mRNAs were all experimentally validated to be overexpressed in either STAD tissues or GC tissues, and the aberrant expression of the remaining F2 in this cancer has not been confirmed by any previous experiments or bioinformatics analysis.

**TABLE 1:** Comparison of the abnormal expressions of the mRNAs-PRO in the overall-, early-, and progressive-stage STAD between this study and previous experimental studies.

Stage	mRNA-PRO	Feature	Abstract	Citation
Overall	EGF	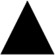	Using western blotting and immunocytochemical staining, Han et al. revealed that compared with normal gastric tissues, EGF was significantly higher expressed in STAD tissues and facilitated the migration and invasion of STAD cells by activating ERK1/2	PMID: 24789460 Han et al. (2014)
	SERPINE1	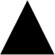	Through the analysis of clinical samples, TCGA database samples, and cell lines *in vitro*, Yang et al. manifested that SERPINEI was significantly upregulated in STAD, and it promoted the invasion, migration, and proliferation of STAD cells by regulating EMT	PMID: 31724495 Yang et al. (2019)
	ESCO2	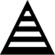	In *in vitro* cell experiments, Chen et al. found that ESCO2 was significantly upregulated in GC cells, and the knockdown of ESCO2 inhibited the proliferation and induced the apoptosis of GC cells	PMID: 29330052 Chen et al. (2018)
	ERCC6L	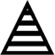	Using qRT-PCR and IHC detections, Chen et al. discovered that compared with normal gastric tissues, ERCC6L was significantly upregulated in GC, and it facilitated the growth and metastasis of GC cells by activating NF-κB signaling	PMID: 34425559 Chen et al. (2021a)
	UHRF1	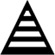	Zhang et al. illustrated that UHRF1 was significantly up-regulated in GC by cell experiments, which further promoted the migration and invasion of the GC cells and inhibited the cell apoptosis through ROS signaling	PMID: 30352833 Zhang et al. (2018)
	SPARC	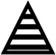	By the analysis of gene microarray data, Wang et al. revealed that SPARC was significantly upregulated in GC cells, and its high expression was positively correlated to the lymph node metastasis, lymphatic infiltration, and shorter survival of GC patients	PMID: 15558074 Wang et al. (2004)
	F2	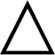	Abnormal expression of F2 has not been reported in any cancer experiments. However, our study showed that F2 was significantly upregulated in STAD, implicating that F2 might be a novel biomarker of STAD	NA
Early	WT1	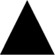	Li et al. found that WT1 was significantly upregulated in STAD by qRT-PCR. In addition, the overexpression of BASP1 in cell experiments of STAD could apparently inhibit the activation of the Wnt/β-catenin pathway to restrain the proliferation, migration, and invasion of STAD cells by downregulating the expression of WT1	PMID: 33426068 Li et al. (2020a)
	JUN	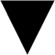	Using IHC, Jin et al. showed that JUN was down-regulated and had tumor suppressor activity in GC. The loss of JUN expression was correlated to a more advanced stage, lymphatic invasion, lymph node metastasis, and shorter survival of GC patients	PMID: 18158,562 Jin et al. (2007)
	IGFBP1	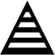	Luo et al. suggested that compared with the control groups, IGFBP1 was significantly upregulated in GC cells infected with *H. pylori* 26695. However, its overexpression could reduce the promoting effect of MMP-9 on the BGC-823 cells migration, indicating the protective role of IGFBP1 in the process of H. pylori-induced GC	PMID: 28864349 Luo et al. (2017)
	APOE	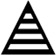	Sakashita et al. suggested *via* RT-PCR that APOE was significantly highly expressed in GC, and GC samples with high expression of APOE had deeper tumor infiltration, more positive lymph node metastasis, and shorter survival compared with low APOE expression patients	PMID: 19020708 Sakashita et al. (2008)
	CA10	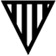	Tao et al. found that CA10 was significantly downregulated in glioma and CA10 secreted by depolarized cultured neurons blocked the neuronal activity-dependent growth and inhibited the invasion of glioma by cell experiments	PMID: 30636076 Tao et al. (2019)
	GRIA2	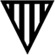	Through qRT-PCR and IHC, Choi et al. manifested that GRIA2 was significantly downregulated in advanced ovarian serous adenocarcinomas and the upregulation of GRIA2 was associated with the better survival of patients	PMID 22644307 Choi et al. (2012)
	TF	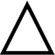	The abnormal expression of TF has not been reported in any cancer experiments. However, our study showed that TF was not differentially expressed in overall-stage STAD, but was significantly up-regulated in the early-stage STAD, implying that TF might be a novel biomarker of early-stage STAD	NA
	GRID2	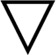	Abnormal expression of GRID2 has not been reported in any cancer experiments. However, Chen et al. suggested that GRID2 was significantly downregulated in endometrial carcinoma, and its expression was significantly associated with the proliferation and invasion of cancer cells through bioinformatics analysis. In this study, it was shown that GRID2 was significantly downregulated in the overall- and early-stage STAD, indicating that GRID2 might be a novel biomarker of these two stages of STAD	PMID: 33577492 Chen et al. (2021c)
	APOB	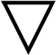	Abnormal expression of APOB has not been reported in any cancer experiments. However, our study showed that APOB was significantly downregulated in the overall- and early- stage STAD, indicating that APOB might be a novel biomarker of these two stages STAD	NA
Progressive	CHAF1A	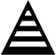	Zheng et al. found that CHAF1A was highly expressed in GC cells and could promote gastric carcinogenesis by upregulating c-MYC and CCND1. Moreover, the overexpression of CHAF1A in progressive-stage STAD were verified by qRT-PCR and IHC in our study	PMID: 30449701 Zheng et al. (2018)
	VCAN	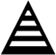	Using qRT-PCR of clinical tissue samples, Cheng et al. illustrated that VCAN was significantly upregulated, which promoted the proliferation, invasion, and migration of GC cells	PMID: 33116649 Cheng et al. (2020)
	CDH11	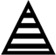	By bioinformatics analysis and cell experiments, Liu et al. found that CDH11 was highly expressed in GC and as an oncogene, CDH11 facilitated GC progression *via* transcriptional up-regulation by HEYL, but its overexpression could reversely promote the malignant behavior of HEYL-knockdown GC cells	PMID: 32463580 Liu et al. (2020)
	PDGFRB	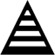	Through the RT-qPCR analysis of clinical tissue samples, Higuchi et al. revealed that PDGFRB was significantly upregulated in stage II/III gastric cancer and was closely related to the poor 5-year survival rate and proliferation of cancer cells	PMID: 28356977 Higuchi et al. (2017)
	BMP1	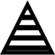	Through targeted RNA sequencing of clinical specimens, Hsieh et al. found that BMP1 was highly expressed in late-stage GC and significantly related to the poor prognosis of GC patients. In addition, *in vitro* suppression of BMP1 led to the reduction of the mobility of the GC cell lines, implying an important role of BMP1 in metastasis	PMID: 29720137 Hsieh et al. (2018)
	ASPN	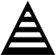	Via IHC assay, Zhang et al. illustrated that ASPN was significantly upregulated in GC, which was related to the poor prognosis of GC patients. The upregulation of ASPN promoted the growth of GC cells and inhibited apoptosis *via* deactivating LEF1-gene transcription independent of β-catenin *in vitro* and *in vivo*	PMID: 34127813 Zhang et al. (2021b)
	BGN	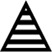	Via *in vitro* and *in vivo* experiments, Pinto demonstrated that BGN was significantly overexpressed in GC tissues, which was related to disease recurrence and poor prognosis of patients with advanced GC. The over-expressed BGN promoted the cell migration, invasion, and angiogenesis of GC	PMID: 33809543 Pinto et al. (2021)
	GINS4	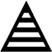	Using the qRT-PCR and IHC detection of clinical tissue samples, Zhu et al. illustrated that GINS4 was significantly overexpressed in GC, and the up-regulation of GINS4 was associated with poor differentiation, advanced stage, depth of invasion, and lymph node metastasis of GC tissues	PMID: 31754397 Zhu et al. (2019)
	COL6A3	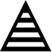	Sun et al. showed that COL6A3 was highly expressed in GC by using RT-PCR, and the overexpression of COL6A3 facilitated the proliferation, migration, and inhibited the apoptosis of GC cells.	PMID: 31122696 Sun et al. (2019)
	ECT2	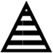	Through RT-qPCR and Western blotting, Zhang et al. suggested that compared with normal control, the expression level of ECT2 in GC increased significantly and was positively correlated to histological differentiation, lymph node metastasis, and TNM stage of GC	PMID: 34367280 Zhang et al. (2021a)
	IBSP	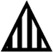	Using the qRT-PCR and IHC detection of clinical tissue samples, Wang et al. suggested that IBSP was overexpressed in esophageal squamous cell carcinoma and the upregulation of IBSP was significantly associated with the lymph node metastasis, clinicopathological stage, and poor disease survival	PMID: 31709184 Wang et al. (2019)
	KIF18B	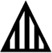	With the qRT-PCR and IHC detection of clinical tissue samples, Yang et al. illustrated that KIF18B was significantly upregulated in hepatocellular carcinoma, thus promoting the progression of hepatocellular carcinoma by activating the Wnt/β-catenin pathway	PMID: 32052444 Yang et al. (2020)
	INCENP	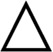	Abnormal expression of INCENP has not been reported in any cancer experiments. However, our study showed that INCENP was not differentially expressed in overall-stage STAD but was significantly upregulated in progressive-stage STAD, indicating that INCENP might be a novel biomarker of progressive-stage STAD	NA
	GCG	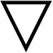	Abnormal expression of GCG has not been reported in any cancer experiments. However, our study showed that GCG was not differentially expressed in overall-stage STAD but was significantly downregulated in the progressive-stage STAD, indicating that GCG might be a novel biomarker of progressive-stage STAD	NA

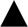
mRNAs experimentally up-regulated in STAD in accordance with our calculated result. 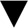
mRNAs experimentally downregulated in STAD in accordance with our calculated result. 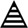
mRNAs experimentally upregulated in GC in accord with our calculated result. 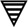
mRNAs experimentally downregulated in GC in accordance with our calculated result. 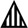
mRNAs experimentally upregulated in other cancers in accordance with our calculated result. 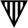
mRNAs experimentally down-regulated in other cancers in accordance with our calculated result. 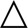
mRNAs upregulated in our calculated results not experimentally verified in cancers. 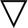
mRNAs downregulated in our calculated results not experimentally verified in cancers.

Among the nine mRNAs in the prognostic model of early-stage STAD, four mRNAs were up-regulated (WT1, IGFBP1, APOE, and TF) and five mRNAs were down-regulated (JUN, CA10, GRIA2, GRID2, and APOB). After comparing the outcomes in the related literature with the findings in our results, it was shown that the high expression level of WT1, IGFBP1, and APOE, and the low expression level of JUN was verified by previous clinical cases or experiments on GC, and the down-regulations of CA10 and GRIA2 were demonstrated in other tumor origins, where the results were all matched with our calculations. Although the remaining three mRNAs have not been reported by any cancer experiments before, GRID2 was calculated to be significantly down-regulated in endometrial carcinoma by bioinformatics studies, and TF and APOB have not been mentioned by any cancer studies before and were novel candidate prognostic signatures in early-stage STAD reported for the first time by this study. Notably, Kalantari et al. demonstrated that Lgr5High/DCLK1 high phenotype was associated with early-stage gastric carcinoma specimens (Kalantari et al., 2017). However, in this study, we found that Lgr5 was significantly upregulated, but DCLK1 was significantly downregulated in early-stage STAD (Supplementary Table S3); moreover, DCLK1 was one of the mRNAs-OS (Supplementary Table S5), though it was not in the 9-mRNA prognostic model of this premature stage. The contradiction between these two studies needs to be further explored.

Among the 14 mRNAs in the prognostic model of progressive-stage STAD, 13 mRNAs (CHAF1A, VCAN, CDH11, PDGFRB, BMP1, ASPN, BGN, GINS4, COL6A3, ECT2, IBSP, KIF18B, and INCENP) were upregulated, while one mRNA (GCG) was downregulated. As shown by the summaries of earlier studies in Table 1, we noted that the overexpression of eleven mRNAs (CHAF1A, VCAN, CDH11, PDGFRB, BMP1, ASPN, BGN, GINS4, COL6A3, ECT2, and IBSP) were all verified in GC cell lines; the increased expression level of KIF18B had been confirmed in hepatocellular carcinoma cells, and INCENP and GCG have not been mentioned by any cancer studies before and were novel candidate prognostic signatures in progressive-stage STAD reported for the first time by this study.

Emerging predictive tools for GC had been established based on prognostic genes (Chen J. et al., 2021b), lymph node histopathology (Wang X. et al., 2021b), immune microenvironment or/and tumor microenvironment-relevant genes (Cai et al., 2020; Wan et al., 2020), deep learning radiomic nomogram (Dong et al., 2020), lncRNAs (Ma et al., 2020), and other scoring systems (Zhang et al., 2019). Although these prognostic models mentioned earlier could predict the outcomes of certain GC patients in diverse clinical practices, they all have shortcomings: they did not be classified as histopathology types or stages, and AUC values of ROC curves for early- and progressive-stage STAD in this study were superior. This demonstrated better survival prediction, probably owing to the more refined and representative data after stratification. Moreover, three models were further confirmed reliably in various clinical circumstance, including different ages (≤65 years or > 65 years), genders (males or females), T stages (T1, 2 or T3, 4), N stages (N0 or N1, 2), and M stage (M0) (Supplementary Figures S1–3), indicating the applicability of prognostic models established by stratified data according to vital clinical variables. As experimental data were enhanced after stratification, novel molecular biomarkers would be identified to facilitate more accurate predictions.

Enrichment analysis of the overall STAD stage was based on mRNAs-OS, whereas the GSEA of stratification stages was based on all mRNAs. As for GO analysis, most of mRNAs-OS were enriched in cell communication, cell growth and/or maintenance, signal transduction, receptor activity, cell adhesion molecule activity, and auxiliary transport protein activity (Figure 5A), which were basic, common, and essential for survival of cancer cells. Both GO and KEGG analyses in stratified STAD stages revealed notable heterogeneity in early- and progressive-stage STAD. In early-stage STAD, GSEA-revealed chemical stimulus, including detection, and sensory were markedly enriched (Figure 6A), but few reports focused on these respects. MRNAs of progressive-stage STAD from GSEA were mainly enriched in immune-related functions, including regulation of lymphocyte activation, regulation of T-cell activation, immunological synapse, cytokine receptor activity, and immune receptor activity (Figures 6B,D,F). Immune-related signatures took part in formation of the tumor microenvironment and could predict prognosis in GC (Dai et al., 2021). Wang et al. performed pathway enrichment analysis based on single-cell sequencing in GC, and 12 pathways were immune-related, such as defensins, IL-7 signaling, and IL6/JAK/STAT3 signaling; meanwhile, these pathways were all associated with longer survival, suggesting certain immune-related biological processes contribute to distinct molecular consequences and patient survival (Wang R. et al., 2021a). As for KEGG analyses in early-stage STAD, regulation of autophagy is of note (Figure 7A). Autophagy is a double-edged sword in GC. On the one hand, it inhibits tumor initiation at early-stage by clearing damaged mitochondria, peroxisome, and also meets the high metabolic needs from enhanced proliferating tumors (Kongara and Karantza, 2012). On the other hand, autophagy protects some tumor cells against nutrient and oxygen deprivation, and harsh tumor microenvironments; meanwhile, autophagy is shown to facilitate resistance to cisplatin in GC cells, and thus, it is a cause of tumor metastasis, recurrence, and chemoresistance (Maes et al., 2013; Katheder et al., 2017). As for KEEG analysis in progressive-stage STAD, cell adhesion molecules (CAMs) were of note (Figure 7B). Cell adhesion molecules have multifaceted roles, including signaling molecules, and key constituents of the cell migration machinery, which are involved in virtually each step of tumor progression from primary cancer development to metastasis (Hamidi and Ivaska, 2018). Altered expression of cell adhesion molecules is frequently detected in tumors, and meanwhile, these molecules contribute to supporting the oncogenic growth factor receptor (GFR) signaling, and GFR-dependent cell invasion and migration, which are main features of progressive tumors (Hamidi and Ivaska, 2018).

Further qPCR and IHC detections were performed to estimate mRNA and protein levels of two representative targets in clinical samples. IGFBP-1, which belongs to the insulin-like growth factor system, plays an essential role in the pathophysiology of various tumors (Lin et al., 2021). The role of IGFBP1 had been explored by previous experiments. Yuya Sato et al. reported IGFBP1 could predict hematogenous metastasis in patients with gastric cancer (Sato et al., 2019), while another research demonstrated that the role of IL35 in GC angiogenesis was altering TIMP1, PAI1, and IGFBP1 (Li X. et al., 2020b). In our study, the experimental results of IGFBP1 in both transcriptional and protein levels were in accordance with the bioinformatics outcome, which illustrated it could predict early-stage STAD. CHAF1A, as a known histone chaperone, is also upregulated in tumors of many origins, including GC. It has been reported CHAF1A could upregulate the c-MYC and CCND1 expressions and further promote gastric carcinogenesis (Zheng et al., 2018). In this study, both qPCR and IHC results of CHAF1A were in line with bioinformatics analysis, which illustrated that it could predict progressive-stage STAD. Thus, these two targets could serve as potential signatures for identification of STAD at different risks.

This study stratified STAD into early and progressive stages, which is closely related to distinct treatment and prognosis, and by combining TCGA data, conclusions drawn are more general and representative. In addition, through research validation in clinical cases, we believed our results were stable and reliable. Meanwhile, it is worth noting that limited to TCGA data, the number of paraneoplastic tissues was small, and cancer and paraneoplastic normal cases were evidently not one-to-one matching. Therefore, selection bias is hard to avoid in bioinformatics results when based on these data. Also, since there were only 30 cases in the qPCR validation cohort, and most of which were progressive-stage STAD, and for IHC, though validated by more than 80 paired STAD and paraneoplastic normal tissues, it was a semi-quantitative method, so subjectivity cannot be ruled out when evaluation of targets. Therefore, further investigations by large and matched cohorts were strongly suggested to be performed in the future.

In conclusion, our study combined bioinformatics analysis, clinical parameters, qPCR, and IHC detections in clinical samples to draw the conclusion that mRNA-based models by stratification could predict outcomes of patients at different risks, and selected signatures could serve as novel biomarkers for STAD patients at varied stages.

## Data Availability

The original contributions presented in the study are included in the article/Supplementary Material; further inquiries can be directed to the corresponding authors.

## References

[B1] Ajani J. A. D'Amico T. A. Almhanna K. Bentrem D. J. Chao J. Das P. (2016). Gastric cancer, version 3.2016, NCCN clinical practice guidelines in oncology. J. Natl. Compr. Canc. Netw. 14 (10), 1286–1312. 10.6004/jnccn.2016.0137 27697982

[B2] Ajani J. A. Lee J. Sano T. Janjigian Y. Y. Fan D. Song S. (2017). Gastric adenocarcinoma. Nat. Rev. Dis. Prim. 3, 17036. 10.1038/nrdp.2017.36 28569272

[B3] Cai W. Y. Dong Z. N. Fu X. T. Lin L. Y. Wang L. Ye G. D. (2020). Identification of a tumor microenvironment-relevant gene set-based prognostic signature and related therapy targets in gastric cancer. Theranostics 10 (19), 8633–8647. 10.7150/thno.47938 32754268 PMC7392024

[B4] Chen D. Liu Q. Cao G. (2021a). ERCC6L promotes cell growth and metastasis in gastric cancer through activating NF-κB signaling. Aging (Albany NY) 13 (16), 20218–20228. 10.18632/aging.203387 34425559 PMC8436930

[B5] Chen H. Zhang L. He W. Liu T. Zhao Y. Chen H. (2018). ESCO2 knockdown inhibits cell proliferation and induces apoptosis in human gastric cancer cells. Biochem. Biophys. Res. Commun. 496 (2), 475–481. 10.1016/j.bbrc.2018.01.048 29330052

[B6] Chen J. Wang A. Ji J. Zhou K. Bu Z. Lyu G. (2021b). An innovative prognostic model based on four genes in asian patient with gastric cancer. Cancer Res. Treat. 53 (1), 148–161. 10.4143/crt.2020.424 32878427 PMC7812008

[B7] Chen X. Zhang W. Zhu H. Lin F. (2021c). Development and validation of a 5-gene autophagy-based prognostic index in endometrial carcinoma. Med. Sci. Monit. 27, e928949. 10.12659/msm.928949 33577492 PMC7885295

[B8] Cheng Y. Sun H. Wu L. Wu F. Tang W. Wang X. (2020). VUp-regulation of VCAN promotes the proliferation, invasion and migration and serves as a biomarker in gastric cancer. Onco. Targets. Ther. 13, 8665–8675. 10.2147/ott.S262613 32922041 PMC7457828

[B9] Choi C. H. Choi J. J. Park Y. A. Lee Y. Y. Song S. Y. Sung C. O. (2012). Identification of differentially expressed genes according to chemosensitivity in advanced ovarian serous adenocarcinomas: Expression of GRIA2 predicts better survival. Br. J. Cancer 107 (1), 91–99. 10.1038/bjc.2012.217 22644307 PMC3389416

[B10] Dai S. Liu T. Liu X. Q. Li X. Y. Xu K. Ren T. (2021). Identification of an immune-related signature predicting survival risk and immune microenvironment in gastric cancer. Front. Cell Dev. Biol. 9, 687473. 10.3389/fcell.2021.687473 34805135 PMC8596572

[B11] Dong D. Fang M. J. Tang L. Shan X. H. Gao J. B. Giganti F. (2020). Deep learning radiomic nomogram can predict the number of lymph node metastasis in locally advanced gastric cancer: An international multicenter study. Ann. Oncol. 31 (7), 912–920. 10.1016/j.annonc.2020.04.003 32304748

[B12] Fu T. Ji X. Bu Z. Zhang J. Wu X. Zong X. (2020). Identification of key long non-coding RNAs in gastric adenocarcinoma. Cancer Biomark. 27 (4), 541–553. 10.3233/cbm-192389 32176636 PMC12662306

[B13] Hamidi H. Ivaska J. (2018). Every step of the way: Integrins in cancer progression and metastasis. Nat. Rev. Cancer 18 (9), 533–548. 10.1038/s41568-018-0038-z 30002479 PMC6629548

[B14] Han J. Xie Y. Lan F. Yu Y. Liu W. Chen J. (2014). Additive effects of EGF and IL-1β regulate tumor cell migration and invasion in gastric adenocarcinoma via activation of ERK1/2. Int. J. Oncol. 45 (1), 291–301. 10.3892/ijo.2014.2401 24789460

[B15] Higuchi A. Oshima T. Yoshihara K. Sakamaki K. Aoyama T. Suganuma N. (2017). Clinical significance of platelet-derived growth factor receptor-β gene expression in stage II/III gastric cancer with S-1 adjuvant chemotherapy. Oncol. Lett. 13 (2), 905–911. 10.3892/ol.2016.5494 28356977 PMC5351282

[B16] Hsieh Y. Y. Tung S. Y. Pan H. Y. Yen C. W. Xu H. W. Deng Y. F. (2018). Upregulation of bone morphogenetic protein 1 is associated with poor prognosis of late-stage gastric Cancer patients. BMC Cancer 18 (1), 508. 10.1186/s12885-018-4383-9 29720137 PMC5930761

[B17] Huang G. Cui F. Yu F. Lu H. Zhang M. Tang H. (2015). Sirtuin-4 (SIRT4) is downregulated and associated with some clinicopathological features in gastric adenocarcinoma. Biomed. Pharmacother. 72, 135–139. 10.1016/j.biopha.2015.04.013 26054687

[B18] Jiang N. Guo Q. Luo Q. (2022). Inhibition of ITGB1-DT expression delays the growth and migration of stomach adenocarcinoma and improves the prognosis of cancer patients using the bioinformatics and cell model analysis. J. Gastrointest. Oncol. 13 (2), 615–629. 10.21037/jgo-22-233 35557569 PMC9086027

[B19] Jim M. A. Pinheiro P. S. Carreira H. Espey D. K. Wiggins C. L. Weir H. K. (2017). Stomach cancer survival in the United States by race and stage (2001-2009): Findings from the CONCORD-2 study. Cancer 123 (24), 4994–5013. 10.1002/cncr.30881 29205310 PMC5826592

[B20] Jin S. P. Kim J. H. Kim M. A. Yang H. K. Lee H. E. Lee H. S. (2007). Prognostic significance of loss of c-fos protein in gastric carcinoma. Pathol. Oncol. Res. 13 (4), 284–289. 10.1007/bf02940306 18158562

[B21] Joshi S. S. Badgwell B. D. (2021). Current treatment and recent progress in gastric cancer. Ca. Cancer J. Clin. 71 (3), 264–279. 10.3322/caac.21657 33592120 PMC9927927

[B22] Kalantari E. Asadi Lari M. H. Roudi R. Korourian A. Madjd Z. (2017). Lgr5High/DCLK1High phenotype is more common in early stage and intestinal subtypes of gastric carcinomas. Cancer Biomark. 20 (4), 563–573. 10.3233/cbm-170383 28946555

[B23] Katheder N. S. Khezri R. O'Farrell F. Schultz S. W. Jain A. Rahman M. M. (2017). Microenvironmental autophagy promotes tumour growth. Nature 541 (7637), 417–420. 10.1038/nature20815 28077876 PMC5612666

[B24] Kongara S. Karantza V. (2012). The interplay between autophagy and ROS in tumorigenesis. Front. Oncol. 2, 171. 10.3389/fonc.2012.00171 23181220 PMC3502876

[B25] Li L. Meng Q. Li G. Zhao L. (2020a). BASP1 suppresses cell growth and metastasis through inhibiting wnt/β-catenin pathway in gastric cancer. Biomed. Res. Int. 2020, 8628695. 10.1155/2020/8628695 33426068 PMC7775134

[B26] Li L. Zhu Z. Zhao Y. Zhang Q. Wu X. Miao B. (2019). FN1, SPARC, and SERPINE1 are highly expressed and significantly related to a poor prognosis of gastric adenocarcinoma revealed by microarray and bioinformatics. Sci. Rep. 9 (1), 7827. 10.1038/s41598-019-43924-x 31127138 PMC6534579

[B27] Li X. Niu N. Sun J. Mou Y. He X. Mei L. (2020b). IL35 predicts prognosis in gastric cancer and is associated with angiogenesis by altering TIMP1, PAI1 and IGFBP1. FEBS Open Bio 10 (12), 2687–2701. 10.1002/2211-5463.13005 PMC7714063 33064893

[B28] Li Y. Feng A. Zheng S. Chen C. Lyu J. (2022). Recent estimates and predictions of 5-year survival in patients with gastric cancer: A model-based period analysis. Cancer Control. 29, 10732748221099227. 10.1177/10732748221099227 35499497 PMC9067041

[B29] Lin Y. W. Weng X. F. Huang B. L. Guo H. P. Xu Y. W. Peng Y. H. (2021). IGFBP-1 in cancer: Expression, molecular mechanisms, and potential clinical implications. Am. J. Transl. Res. 13 (3), 813–832. 33841624 PMC8014352

[B30] Liu H. Ni S. Wang H. Zhang Q. Weng W. (2020). Charactering tumor microenvironment reveals stromal-related transcription factors promote tumor carcinogenesis in gastric cancer. Cancer Med. 9 (14), 5247–5257. 10.1002/cam4.3133 32463580 PMC7367614

[B31] Lu Z. Luo T. Pang T. Du Z. Yin X. Cui H. (2019). MALAT1 promotes gastric adenocarcinoma through the MALAT1/miR-181a-5p/AKT3 axis. Open Biol. 9 (9), 190095. 10.1098/rsob.190095 31480991 PMC6769293

[B32] Luo C. Sun F. Zhu H. Ni Y. Fang J. Liu Y. (2017). Insulin-like growth factor binding protein-1 (IGFBP-1) upregulated by *Helicobacter pylori* and is associated with gastric cancer cells migration. Pathol. Res. Pract. 213 (9), 1029–1036. 10.1016/j.prp.2017.08.009 28864349

[B33] Ma B. Li Y. Ren Y. (2020). Identification of a 6-lncRNA prognostic signature based on microarray re-annotation in gastric cancer. Cancer Med. 9 (1), 335–349. 10.1002/cam4.2621 31743579 PMC6943089

[B34] Maes H. Rubio N. Garg A. D. Agostinis P. (2013). Autophagy: Shaping the tumor microenvironment and therapeutic response. Trends Mol. Med. 19 (7), 428–446. 10.1016/j.molmed.2013.04.005 23714574

[B35] Pinto F. Santos-Ferreira L. Pinto M. T. Gomes C. Reis C. A. (2021). The extracellular small leucine-rich proteoglycan biglycan is a key player in gastric cancer aggressiveness. Cancers (Basel) 13 (6), 1330. 10.3390/cancers13061330 33809543 PMC8001774

[B36] Qiu J. Sun M. Wang Y. Chen B. (2020). Identification of hub genes and pathways in gastric adenocarcinoma based on bioinformatics analysis. Med. Sci. Monit. 26, e920261. 10.12659/msm.920261 32058995 PMC7034404

[B37] Ren C. Wu C. Wang N. Lian C. Yang C. (2021). Clonal architectures predict clinical outcome in gastric adenocarcinoma based on genomic variation, tumor evolution, and heterogeneity. Cell Transpl. 30, 963689721989606. 10.1177/0963689721989606 PMC8085378 33900127

[B38] Ren N. Liang B. Li Y. (2020). Identification of prognosis-related genes in the tumor microenvironment of stomach adenocarcinoma by TCGA and GEO datasets. Biosci. Rep. 40 (10), BSR20200980. 10.1042/bsr20200980 33015704 PMC7560520

[B39] Rihawi K. Ricci A. D. Rizzo A. Brocchi S. Marasco G. Pastore L. V. (2021). Tumor-associated macrophages and inflammatory microenvironment in gastric cancer: Novel translational implications. Int. J. Mol. Sci. 22 (8), 3805. 10.3390/ijms22083805 33916915 PMC8067563

[B40] Sakashita K. Tanaka F. Zhang X. Mimori K. Kamohara Y. Inoue H. (2008). Clinical significance of ApoE expression in human gastric cancer. Oncol. Rep. 20 (6), 1313–1319. 19020708

[B41] Sato Y. Inokuchi M. Takagi Y. Kojima K. (2019). IGFBP1 is a predictive factor for haematogenous metastasis in patients with gastric cancer. Anticancer Res. 39 (6), 2829–2837. 10.21873/anticanres.13411 31177120

[B42] Sun X. Zhang X. Zhai H. Zhang D. Ma S. (2019). A circular RNA derived from COL6A3 functions as a ceRNA in gastric cancer development. Biochem. Biophys. Res. Commun. 515 (1), 16–23. 10.1016/j.bbrc.2019.05.079 31122696

[B43] Sung H. Ferlay J. Siegel R. L. Laversanne M. Soerjomataram I. Jemal A. (2021). Global cancer Statistics 2020: GLOBOCAN estimates of incidence and mortality worldwide for 36 cancers in 185 countries. Ca. Cancer J. Clin. 71 (3), 209–249. 10.3322/caac.21660 33538338

[B44] Tao B. Ling Y. Zhang Y. Li S. Zhou P. Wang X. (2019). CA10 and CA11 negatively regulate neuronal activity-dependent growth of gliomas. Mol. Oncol. 13 (5), 1018–1032. 10.1002/1878-0261.12445 30636076 PMC6487704

[B45] Wan L. Tan N. Zhang N. Xie X. (2020). Establishment of an immune microenvironment-based prognostic predictive model for gastric cancer. Life Sci. 261, 118402. 10.1016/j.lfs.2020.118402 32926930

[B46] Wang C. S. Lin K. H. Chen S. L. Chan Y. F. Hsueh S. (2004). Overexpression of SPARC gene in human gastric carcinoma and its clinic-pathologic significance. Br. J. Cancer 91 (11), 1924–1930. 10.1038/sj.bjc.6602213 15558074 PMC2409771

[B47] Wang M. Liu B. Li D. Wu Y. Wu X. Jiao S. (2019). Upregulation of IBSP expression predicts poor prognosis in patients with esophageal squamous cell carcinoma. Front. Oncol. 9, 1117. 10.3389/fonc.2019.01117 31709184 PMC6823256

[B48] Wang R. Dang M. Harada K. Han G. Wang F. Pool Pizzi M. (2021a). Single-cell dissection of intratumoral heterogeneity and lineage diversity in metastatic gastric adenocarcinoma. Nat. Med. 27 (1), 141–151. 10.1038/s41591-020-1125-8 33398161 PMC8074162

[B49] Wang X. Chen Y. Gao Y. Zhang H. Guan Z. Dong Z. (2021b). Predicting gastric cancer outcome from resected lymph node histopathology images using deep learning. Nat. Commun. 12 (1), 1637. 10.1038/s41467-021-21674-7 33712598 PMC7954798

[B50] Wu M. Xia Y. Wang Y. Fan F. Li X. Song J. (2020). Development and validation of an immune-related gene prognostic model for stomach adenocarcinoma. Biosci. Rep. 40 (10), BSR20201012. 10.1042/bsr20201012 33112406 PMC7593539

[B51] Xia N. Xia L. Zhang W. F. Zhou F. X. (2022). Immune-related genes and their determined immune cell microenvironment to predict the prognosis of gastric adenocarcinoma. Zhonghua Yi Xue Za Zhi 102 (12), 840–846. 10.3760/cma.j.cn112137-20211023-02348 35330576

[B52] Yang B. Wang S. Xie H. Wang C. Gao X. Rong Y. (2020). KIF18B promotes hepatocellular carcinoma progression through activating Wnt/β-catenin-signaling pathway. J. Cell. Physiol. 235 (10), 6507–6514. 10.1002/jcp.29444 32052444

[B53] Yang J. D. Ma L. Zhu Z. (2019). SERPINE1 as a cancer-promoting gene in gastric adenocarcinoma: Facilitates tumour cell proliferation, migration, and invasion by regulating EMT. J. Chemother. 31 (7-8), 408–418. 10.1080/1120009x.2019.1687996 31724495

[B54] Yue T. Zuo S. Zhu J. Guo S. Huang Z. Li J. (2021). Two similar signatures for predicting the prognosis and immunotherapy efficacy of stomach adenocarcinoma patients. Front. Cell Dev. Biol. 9, 704242. 10.3389/fcell.2021.704242 34414187 PMC8369372

[B55] Zhang D. He W. Wu C. Tan Y. He Y. Xu B. (2019). Scoring system for tumor-infiltrating lymphocytes and its prognostic value for gastric cancer. Front. Immunol. 10, 71. 10.3389/fimmu.2019.00071 30761139 PMC6361780

[B56] Zhang H. Geng Y. Sun C. Yu J. (2021a). Upregulation of ECT2 predicts adverse clinical outcomes and increases 5-fluorouracil resistance in gastric cancer patients. J. Oncol. 2021, 2102890. 10.1155/2021/2102890 34367280 PMC8337122

[B57] Zhang H. Song Y. Yang C. Wu X. (2018). UHRF1 mediates cell migration and invasion of gastric cancer. Biosci. Rep. 38 (6), BSR20181065. 10.1042/bsr20181065 30352833 PMC6435548

[B58] Zhang Z. Min L. Li H. Chen L. Zhao Y. Liu S. (2021b). Asporin represses gastric cancer apoptosis via activating LEF1-mediated gene transcription independent of β-catenin. Oncogene 40 (27), 4552–4566. 10.1038/s41388-021-01858-7 34127813

[B59] Zhao R. Peng C. Song C. Zhao Q. Rong J. Wang H. (2020). BICC1 as a novel prognostic biomarker in gastric cancer correlating with immune infiltrates. Int. Immunopharmacol. 87, 106828. 10.1016/j.intimp.2020.106828 32736193

[B60] Zheng L. Liang X. Li S. Li T. Shang W. Ma L. (2018). CHAF1A interacts with TCF4 to promote gastric carcinogenesis via upregulation of c-MYC and CCND1 expression. EBioMedicine 38, 69–78. 10.1016/j.ebiom.2018.11.009 30449701 PMC6306399

[B61] Zhou L. Huang W. Yu H. F. Feng Y. J. Teng X. (2020). Exploring TCGA database for identification of potential prognostic genes in stomach adenocarcinoma. Cancer Cell Int. 20, 264. 10.1186/s12935-020-01351-3 32581654 PMC7310509

[B62] Zhu Z. Yu Z. Rong Z. Luo Z. Zhang J. Qiu Z. (2019). The novel GINS4 axis promotes gastric cancer growth and progression by activating Rac1 and CDC42. Theranostics 9 (26), 8294–8311. 10.7150/thno.36256 31754397 PMC6857050

