# Supplementary figures and images for: An integrated analysis of prognostic mRNA signature in early- and progressive-stage gastric adenocarcinoma
Source: Front Mol Biosci. 2023 Jan 4;9:1022056. doi: 10.3389/fmolb.2022.1022056 (PMC9846543; doi:10.3389/fmolb.2022.1022056)

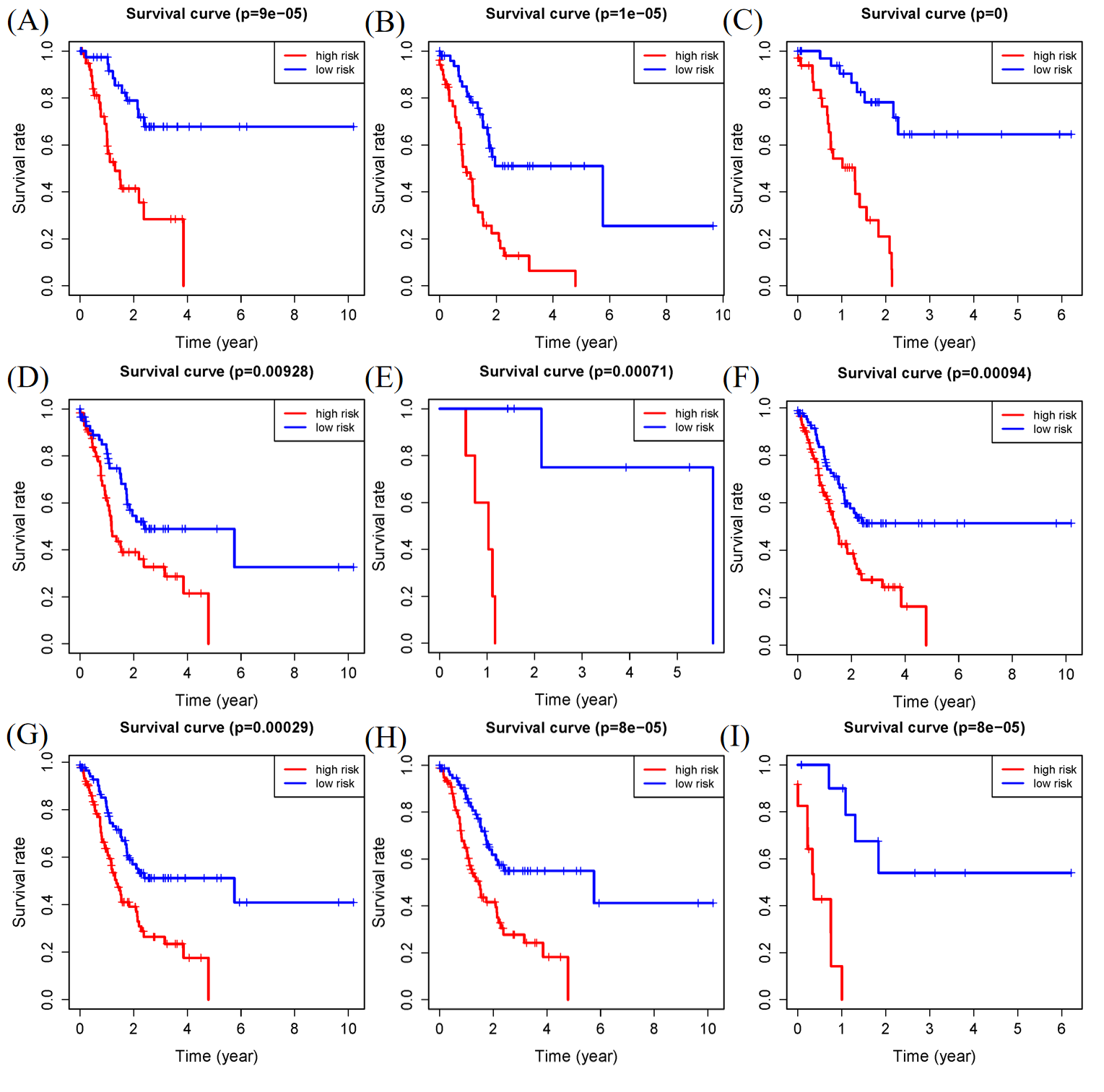

Supplement: Supplementary file 1 [file Image3.TIF]

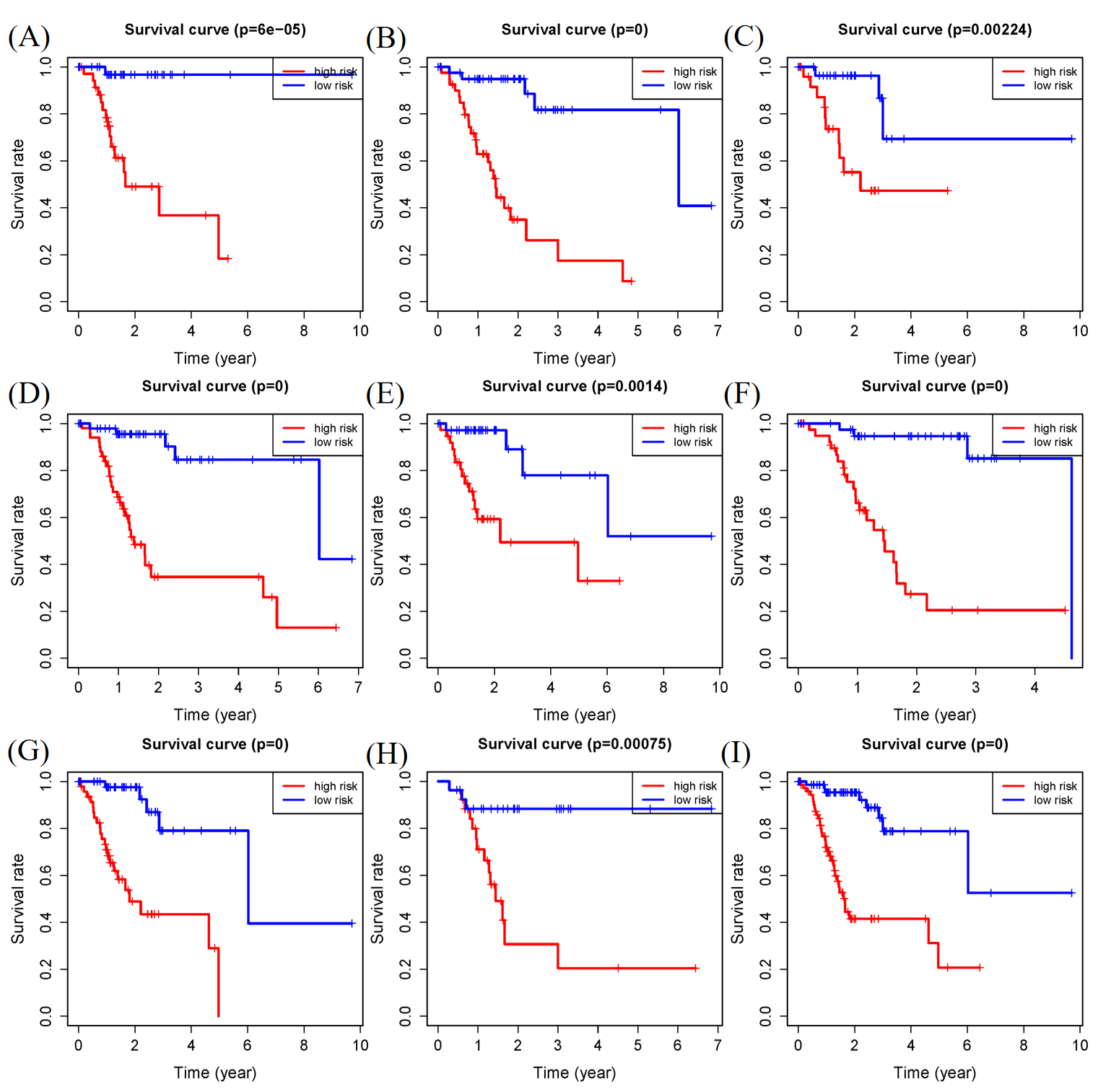

Supplement: Supplementary file 3 [file Image2.TIF]

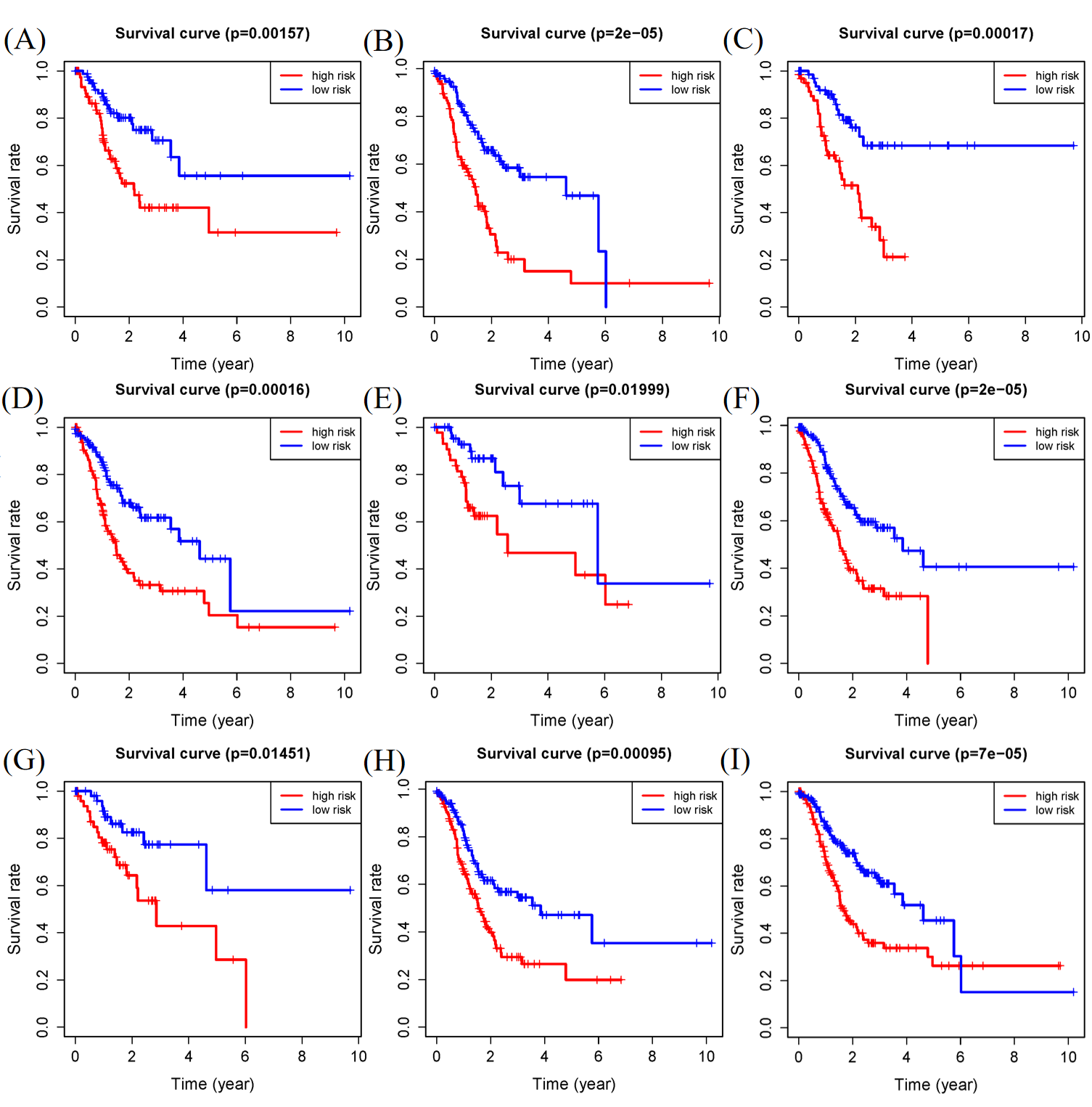

Supplement: Supplementary file 4 [file Image1.TIF]
